# A Survey on Intermediation Architectures for Underwater Robotics

**DOI:** 10.3390/s16020190

**Published:** 2016-02-04

**Authors:** Xin Li, José-Fernán Martínez, Jesús Rodríguez-Molina, Néstor Lucas Martínez

**Affiliations:** Research Center on Software Technologies and Multimedia Systems for Sustainability (Centro de Investigación en Tecnologías Software y Sistemas Multimedia Para la Sostenibilidad—CITSEM), Campus Sur UPM, Ctra. Valencia, Km 7, Madrid 28031, Spain; jf.martinez@upm.es (J.-F.M.); jesus.rodriguezm@upm.es (J.R-M.); nestor.lucas@upm.es (N.L.M.)

**Keywords:** middleware, underwater robotics, Autonomous Underwater Vehicles (AUV), Autonomous Surface Vehicle (ASV), survey

## Abstract

Currently, there is a plethora of solutions regarding interconnectivity and interoperability for networked robots so that they will fulfill their purposes in a coordinated manner. In addition to that, middleware architectures are becoming increasingly popular due to the advantages that they are capable of guaranteeing (hardware abstraction, information homogenization, easy access for the applications above, *etc.*). However, there are still scarce contributions regarding the global state of the art in intermediation architectures for underwater robotics. As far as the area of robotics is concerned, this is a major issue that must be tackled in order to get a holistic view of the existing proposals. This challenge is addressed in this paper by studying the most compelling pieces of work for this kind of software development in the current literature. The studied works have been assessed according to their most prominent features and capabilities. Furthermore, by studying the individual pieces of work and classifying them several common weaknesses have been revealed and are highlighted. This provides a starting ground for the development of a middleware architecture for underwater robotics capable of dealing with these issues.

## 1. Introduction

Underwater robotics are gaining momentum as useful tools when issues and challenges spring up regarding maritime operations: infrastructure maintenance [1], oil spill counter measurements [2] and survey operations [3] are only a few of the use cases that these devices have proven to have a major usability in, to the point that not having them severely jeopardizes achieving the objectives pursued during a mission. Underwater robotics comprises a plethora of different vehicles capable of providing different capabilities, such as Autonomous Underwater Vehicles (AUVs), Autonomous Surface Vehicles (ASVs, working in close cooperation with the former ones) or generally speaking Unmanned Underwater Vehicles (UUVs) that require little input from a human operator to perform underwater-related tasks. While capable of diving to significant depths or retrieving samples by themselves, a plurality of these vehicles are expected to be deployed during a mission, since the incidence that is being dealt with can easily outmatch the capabilities of a single vehicle (for example, an oil platform is too large to be examined by just one underwater one). Although having several robots while carrying out a mission is advisable, it must be taken into account how the data that are obtained will be processed and transferred from the very vehicle that has collected them to an entity where it will be used by human operators or policy makers in order to gather conclusions from them and take actions during an event. This is an issue that must not be ignored, as any malfunction or failure in data management will result in either lacking or misleading information that will be worthless (or even worse, counterproductive) to plan how to better solve a problem as the ones described before.

Fortunately, intermediation architectures can be used in this environment as they would be used in any other circumstance. An intermediation architecture can be conceived as a distributed software entity located between hardware devices and a more application-oriented layer which is capable of collecting information from hardware devices, treating it as required by a system, and transferring it to a remote location where it will be used as a service or part of an application. Intermediation architectures, or *middleware* as they are also called, abstract the heterogeneity of the information obtained from different data producers so that it will result in a homogeneous looking set of facilities that will be consumed at a higher, closer to the end users level, regardless of their role in the deployment (staff, general public, *etc.*). Thus, it becomes clear that proposals for middleware or intermediation architectures focused on underwater robotics become a topic worth paying attention to, especially if they have been tested to be fully functional in a real environment with underwater robots deployments. This paper deals with the most prominent and useful proposals that have been found regarding middleware and intermediation architectures for undersea robotics, highlighting both their strong points and common weaknesses, as well as putting forward how they can be improved to match any other distributed system with a higher degree of development.

### 1.1. The Need for Middleware Architectures in Underwater Heterogeneous Environments

**Figure 1:**
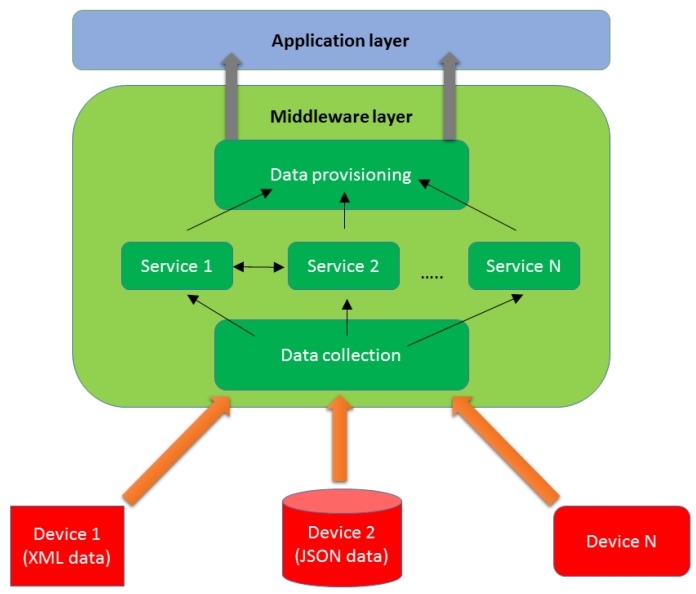
Middleware location in a distributed system.

As stressed before, middleware and/or intermediation architectures become of critical importance when data of different nature, and specifically, of different appearance and syntax, have to be treated in a distributed system. This is an issue that has existed for quite a while, and it precedes the development of effective underwater robotics for years. As a concept, middleware was first mentioned in a NATO report dated back to October 1968 [4]. During the 1980s its popularity and usage increased as a solution to have both new and legacy hardware equipment working in the same network, thus allowing the integration of new appliances and capabilities while at the same time using already existing ones, thus obtaining interoperability and scalability. Middleware guarantees that, despite a lack of standardization regarding how data is transmitted (for example, some appliances may transmit information as JSON messages, others as XML or even raw, as obtained data), the information contained will be extracted, processed and offered to any application or end user that may require it. As can be seen in Figure 1, data are transmitted and provided as a commodity regardless of the appliance they are obtained from.

In addition to that, services can be provided within the middleware layer (context awareness, security, device registration, *etc.*) when they cannot be offered by other elements due to several reasons (data format that cannot be comprehended by other entities without the intervention of intermediation architectures, not enough computational capabilities, *etc.*). In a nutshell, it becomes natural for any deployment that requires data transfer from one entity to another that they will be using some kind of intermediation architecture or middleware layer in order to fulfill data transmissions.

### 1.2. Cooperation and Communication in Underwater Heterogeneous Environments

Due to the nature of their usual tasks, underwater vehicles will be required to cooperate one with the other to fulfill the tasks that are given. Therefore, information will be shared and transferred among them in a sorted way, and taking into account that there are many different vendors selling vehicles of these features, it becomes convenient to have a middleware architecture that will manage data transfers from one deployed entity to the other. It is important to take into account that during this process, communications will indeed be used at a lower, physical level so that data will be transmitted wirelessly, but these data transmission channels are used for byte transmission and are not concerned about topics as data format or information management. Also, since in most cases acoustic waves are used, fast data transmission rates cannot be expected (typical bit rates are in the kilobits range) so data must be transmitted in the most compact possible way so as not to lower system performance.

### 1.3. Related Works

There is an upsurge in exploiting intermediation architectures to abstract the heterogeneity of hardware and ease the development of applications in the robotic field. A great number of middleware frameworks have emerged to facilitate the interaction between robot systems and numerous heterogeneous entities (that usually comprise both software and hardware). It is clear in the reviewed literature that a considerable amount of efforts have been done to collect, analyze, and classify the existing middleware solutions for robotics. [5] indicates a set of challenges residing in the current robotic middleware research. Mohamed *et al.* [6] briefly summarize several research projects working towards the exploitation of middleware in robotics. Furthermore, [7] provides a specific focus on reviewing the study of networked robot-related middleware. [8] gives an overview of the most used robotic middleware architectures by identifying the common concepts and goals and highlighting the lacks. Several open source middleware frameworks for multi-robot systems are listed and elaborated in [9]. In addition to that, Elkady *et al.* [10] provide a very comprehensive literature survey on robotics middleware and make a thorough comparison based on a set of critical attributes, such as architecture, simulation environment, standards and technologies, support for a distributed environment, real-time and behavior cooperation capabilities *etc.* To the best of our knowledge, [10] represents the newest and most extensive work done so far on examining the existing and most popular robotics middleware, including:

- CLARAty [11] is defined as a framework both generic and object-oriented used for new algorithms integration in a variety of fields, such as vision, control, locomotion, navigation, localization, manipulation, planning and execution. It is mentioned to have been adapted to heterogeneous robots with different hardware control architectures and mechanisms.
- Player 2.0 [12], based on a previous work named Player/Stage that uses robotics under a client-server paradigm, is claimed to improve the latter in two aspects: simplicity (withholding some of the features of the driver API from the user) and flexibility (library division to allow its usage with different transport layers).
- ORCA [13] is an open-source software project making use of component-based software engineering to robotics. The framework is described to be the most prominent feature of the proposal, as it is able to adopt a commercial middleware package, an approach towards framework design that intends to be as minimalist as possible, and a stress on multi-language and multi-platform support.
- MIRO [14] is another object-oriented robot middleware focused on obtaining portability and maintainability for the developed robot software. It provides generic abstract services like localization or behavior engines.
- OpenRTM-aist [15] implements some of the extended features that are used according to the Robotics Technology (RT) middleware standard. It has actually been developed by the same institution that created the RT standard (National Institute of Advanced Industrial Science and Technology, Tokyo, Japan). It includes a manager component to aid the manipulation of other RT Components, too.
- ASEBA [16] is described as a modular architecture for event-based control of complex robots. It is used by running script inside virtual machines with self-contained actuators and sensors.
- MARIE [17] can be regarded as a middleware framework used to integrate both new and legacy software in robotic systems. The authors list as a success the development of a “socially interactive autonomous mobile robot platform” able to perform several different functionalities, such as localization, navigation, map building, sound source localization, tasks scheduling, tracking and separation, visual tracking, *etc.*
- RSCA [18], an acronym for Robot Software Communications Architecture, is composed of a real-time operating system, deployment and communication middleware in a hierarchical way. The functionalities that can be used with the middleware modules are creation, installation, start, stop, tear-down and uninstallation.
- OPRoS [19] makes use of a component-based development paradigm to provide a robot software platform to support the development of new software. It offers specifications for several components: model, composer, an authoring tool and an execution engine.
- ROS [20] is a flexible framework used for robotics software development. It provides a set of several facilities (libraries, tools, conventions) aimed to aid the creation of software that will be used across a variety of platforms and coded with several different coding languages. A significant number of the proposals related to underwater robotics that have been studied rely on ROS to an extent for their performance.
- MRDS or Microsoft Robotics Developer Studio [21] is an environment used for robot control and simulation. Since it has been developed by Microsoft, it should come as no surprise that it is aimed to development under Windows-based operating systems. It offers a suite of tools (visual programming, windows and web-based interfaces, *etc.*) to make development possible.

Other proposals that can be taken into account are: GenoM3 [22], Nerve [23] and MIRA [24]. As can be seen, there is extensive literature about intermediation architectures for regular environments. However, the focus of all the aforementioned work is put on the investigation of generic robotic middleware. A major lack detected in the current literature is that none of existing survey papers provides an insightful review on robotic middleware for underwater environments. Due to the increasing needs of applying middleware technologies to integrate and coordinate groups of underwater vehicles, it is of great importance to put an emphasis on reviewing and categorizing existing middleware solutions explicitly devoted to underwater robotics. Therefore, one of the major contributions that have been done in this paper is surveying the state of the art regarding intermediation architectures for robots in underwater environments.

### 1.4. Contributions Offerred

The main contributions made by this manuscript can be summarized as:

- Extraction of the advisable requirements that a middleware architecture should have when it is deployed in an underwater environment, especially when it is done so in underwater robotics.
- A survey of the most prominent proposals has been included, highlighting their most important characteristics and how they are used.
- Open issues that have been found as a result of the study done.
- Future works that can be carried out so that the quality of future middleware architectures in underwater robotics will be improved.

The remaining parts of this paper are organized as follows: Section 2 deals with the features that have been put forward as critical in a middleware architecture used in underwater robotics, which will be used for the assessment of the studied proposals. Section 3 reviews twelve prominent and new underwater middleware architectures along with thorough analyses. Section 4 describes the open issues that have been found among the presented works. These open issues are obtained after taking into account the degree of accomplishment in each of the proposals with regards to the features that were defined in Section 2. Conclusions and future works are presented in Section 5.

## 2. Technical Features for an Underwater Middleware Architecture

According to the information that has been offered in the Introduction, and how it has been studied in the researched proposals regarding intermediation architectures for underwater environments, a collection of features can be expected from a middleware architecture to offer its functionalities in a satisfying way. They can be inferred from the following statements:

1. Middleware must be capable of handling different data formats that might be used by equipment manufactured by different vendors as the ones that based their businesses in robotics or software intermediation architectures. Thus, data heterogeneity management must be considered.
2. Since middleware is used to interconnect pieces of equipment scattered in a relatively wide area (underwater and/or surface vehicles that are deployed during a mission) it has to take into account system distribution.
3. Optimization of the information that is transferred throughout the distributed collection of deployed robots is usually required by the parties involved in the deployment. One of the most efficient methods to get is inferring the knowledge that is contained in the sent and received data. Therefore, semantic capabilities become a desirable way to improve middleware and the deployment where it is located.
4. Middleware is not only used as a messenger between the deployed pieces of equipment, it can also offer some other services, such as device registration, quality of service, security features, *etc.* Consequently, middleware services must also be taken into account.
5. Any intermediation architecture must be capable of carrying on working despite of faults in the hardware appliances where it has been installed (for example, one of the robot sensors takes damage or its control signal is lost for a while). Thus, fault tolerance becomes a feature more than welcome to offer in an architecture like this.
6. As it happens with many other software developments, scalability is also needed, as the middleware that is installed in the underwater robots should be capable of coping with new hardware additions and other appliances involved in missions, due to the cooperative nature of them (several AUVs and/or ASVs may have to be deployed during a mission).
7. Deployed robots must be aware of the different parameters of their surroundings so that their actions can be done in an effective manner, as missions usually involve a degree of knowledge and interaction with the context they have been sent into. Due to this reason, context awareness must be regarded as another major feature in a middleware expected to work with underwater robots.
8. Features as data integrity, confidentiality and user authenticity are important as well so that the transferred information will not be misused by a hostile third party. In a distributed environment, as underwater robotic deployments are, this is a major concern because there are more potential zones where information can be leaked or tarnished. Therefore, security will also be taken into account as a significant feature.
9. Some of the objectives of the missions that are carried out by underwater robots require real time capabilities (conceiving this as the capacity of the system to react to changes that take place at one specific moment almost at that very moment), especially when fast decisions have to be made. Real time as a feature is also addressed in this study.
10. Last but not least, the easiness to provide information to the community of developers and the usage of open source resources must also be commented, as there is an ever-increasing amount of middleware solutions that rely on open source-based tools and developments. All in all, the availability of information can also be a very useful tool for underwater robotics middleware. Therefore, information availability is also included in this study.

As can be inferred from the previous comments, a full-fledged middleware architecture conceived for underwater robotics usage is expected to be capable of satisfying a specific set of features: data heterogeneity management, system distribution, semantic capabilities, system services, fault tolerance, scalability, context awareness, security, real time information transfer and information availability. Consequently, the proposals that have been included in this survey are assessed taking into account that set of features, as they are more comprehensive than any other set that has been found by the authors of this manuscript. However, depending on the orientation of other areas of knowledge, the proposals presented here may fare better or worse in other contexts. Unsurprisingly, there are ten different features that have been defined as critical in order to design an intermediation architecture good enough for the tasks that are expected to be tackled. As a way to have an assessment as objective and accurate as possible, several rubrics have been defined for each of the features that are regarded as essential in the study. The common theme in them is that they provide a score ranging from 1 (very poor fulfillment of the assessed feature) to 5 (very good fulfillment of the assessed feature) through 2 (poor fulfillment), 3 (average fulfillment) and 4 (good fulfillment). Last but not least, when evaluating the proposals, it has to be taken into account that they have been evaluated according to the criteria that are presented in this manuscript. Rather than using a criterion of “good” or “bad”, what is considered here is to what extent the proposal matches the features that have been presented before. Therefore, it should not be inferred that, if a proposal is assessed with a low score then is a low quality one, but that does not really fit with the characteristics that have been presented before, according to the criteria of the authors.

### 2.1. Data Heterogeneity Management

This feature takes into account how data heterogeneity is managed. It is assumed that heterogeneous data will be collected from the underwater environment where the different vehicles and pieces of hardware are deployed. The proposal will be assessed depending on the treatment that is done to the data. Also, it is taken into account the quality of the tests that have been carried out in order to obtain results of the performance regarding the proposal. Commonly, the more prominent an intermediation or middleware architecture is present, the better data can be managed, as the features required are clearly confined within the boundaries of that architecture. Table 1 shows how data heterogeneity management is evaluated.

**Table 1:** Data heterogeneity management assessment.

Architecture Features	Grade Description
Grade: 5	The proposal manages data by means of a differentiated layer that uses a set of very specific and well-defined set of functionalities. The procedure used to perform that data management is described with profuse details. Standards have been used to homogenize data transferred (XML, JSON, common information models, *etc.*). Extensive implementation and testing details are provided with actual deployments rather than simulations whenever it is possible to do so.
Grade: 4	A separated middleware architecture or layer is used for data management. The procedures used to describe data heterogeneity are shown in a very detailed manner. Standards have not been used to homogenize data that is being transferred, so it might be harder to port the middleware architecture to another system. Implementation and testing information is provided in order to know about the performance of the proposal. Some details might be vague or testing might be limited to simulations.
Grade: 3	A layer used for data heterogeneity management is provided in the proposal, although it lacks details or there is not abundant information abut its inner components, messages interchanges or performance results. Heterogeneous data is managed correctly but explanations are not detailed enough.
Grade: 2	Poor information is provided about the proposal and the intermediation architecture that is provided or its inner procedures. Diagrams and illustrations are available, but they provide only hints and vague information.
Grade: 1	There is not a differentiated intermediation architecture layer (such as middleware) to manage data. Information regarding how heterogeneous data are managed is either non-existent or irrelevant.

### 2.2. System Distribution

This feature takes into account how distributed the system is. According to the research that has been put forward in this manuscript, a distributed system offers several advantages over a centralized one, especially if autonomous units of equipment are involved in a system, as it will provide a more effective performance (according to the scope of this paper, communications and commands do not have to be transmitted as frequently as in a centralized system using the AUVs as mere appendixes of a central one, actions and decisions can be taken without waiting for instructions from an external entity, *etc.*). The rubric for the assessment has been depicted in Table 2.

**Table 2:** System distribution assessment.

Architecture Features	Grade Description
Grade: 5	The system works with a large distribution degree, not having any centralized point that can be overloaded to the point that, if collapsing, would jeopardize the performance of the system. Extensive information is provided about how implementation and testing have been carried out, among other stages of the development of the solution, and those stages of development are done using actual pieces of equipment in a real deployment.
Grade: 4	The system is distributed but there is some kind of prominent element within the deployment regarding the distribution of the system, so distribution is not complete and if that prominent element fails, the whole system could be in trouble as it cannot be easily replaced by another node. Testing activities have been done but they are using a simulated environment rather than a real one.
Grade: 3	Information about the distributed nature of the proposal is provided (significant and descriptive illustrations and descriptions are available, message formats are offered, UML diagrams explain the inner performance of the system *etc.*) and it becomes justified how the architecture is a distributed one, but just at a high level without getting into a deep description level. Scarce information is available with regards to the system performance.
Grade: 2	Little information is provided about the distributed nature of the proposal. Hints are put forward on how the system is not centralized.
Grade: 1	There is no data about system distribution (the most prominent functionalities are conceived as centralized), or it is presented with little to no importance or stress.

### 2.3. Semantic Capabilities

In this case it is assessed the degree of semantics that is present in the proposal. Semantics are usually of major importance in order to extract information of the system and be able to infer and learn about the context where the deployment is done. For underwater robotics it is even more important, as AUVs may be put in the situation of having to take decisions by themselves, so a significant degree of semantic capabilities must be provided for them. Table 3 shows how semantic capabilities are considered in the proposals.

**Table 3:** Semantic capabilities assessment.

Architecture Features	Grade Description
Grade: 5	An extensive description of the semantic capabilities of the system is provided: new ontologies have been created for the deployment capable of storing content specific of underwater robots environment (chemical data, temperatures, underwater currents force, *etc.*), or already existing ones become seamlessly adapted for the use cases described in the proposal. Descriptive information regarding implementation and testing of the proposal is provided, especially regarding real or large environments.
Grade: 4	The proposal uses semantic capabilities to a significant extent and information relevant to the mission where the robots have been deployed is inferred from the data collected from the environment. Development or adaptation of new ontologies is provided as available information. Testing of the capabilities of the semantics-related modules is provided at a simulation or laboratory level.
Grade: 3	Semantics are present in the proposal and the functionalities of the ontologies or alike additions are explained, although it is not clear how they are implemented. Adaptation of already existing ontologies has been done but no new ones have been created.
Grade: 2	Scarce details are provided regarding semantics; it is only mentioned that they are present, but without information about to what extent and how they are specifically implemented.
Grade: 1	There is no information about data semantics in the proposal that is being assessed.

### 2.4. System Services

This feature measures the quantity and quality of the features that have been added in an underwater robotics middleware. The services described here have as their common characteristic that are offered from the middleware architecture to its surrounding elements, that is to say, either they will provide a benefit to the lower, hardware-based layer (AUVs, ASVs, *etc.*) or to the applications that are right above this intermediation architecture. By means of these applications, end users will be able to access in a seamless fashion to all the services offered by the middleware architecture. The more the users participate of the system, the higher the benefits provided by the services embedded in the middleware architecture will be.

Commonly, there will be some services that, one way or another, are implemented (equipment or data registration, data transfer from lower to upper levels of the proposal architecture), but there are some others that, while enhancing the proposal overall, are not widely implemented (security, context awareness, decision making modules). Table 4 shows the evaluation of system services.

**Table 4:** System services assessment.

Architecture Features	Grade Description
Grade: 5	A complete plethora of services is provided by the proposal (data registration, decision making modules, security, context awareness, events, upper layer interaction, *etc.*). The implementation of those services is thoroughly described in terms of performance. Furthermore, testing of these services has been done in actual hardware and performance results have been obtained.
Grade: 4	The proposal has a remarkable number of services, taking into account the inputs and the outputs that must be obtained from them. Requirements that resulted in the implementation of those services are described and diagrams are offered. Testing has been done in a simulated environment.
Grade: 3	The set of services that are put forward by the proposal are not fully implemented, although they are fully described, either as part of specifications of the proposal or as a future work that can be done in the future. Some implementation works are shown and an implementation of the available services is offered, but inly to a limitied extent without extensive performance results.
Grade: 2	Poor information is offered regarding the services that can be provided by the proposal. Illustrations and diagrams are available regarding the services to be obtained. Scarce information about services implementation is offered.
Grade: 1	No information about the services that are provided by the proposal is offered or details are too scarce to have a good idea of the services that can be expected from it.

### 2.5. Fault Tolerance

By this feature it is meant how the system is capable of carrying on with its normal performance whenever an error of certain significance happens (one of the involved entities takes damage, communications falter, erroneous data is obtained, *etc.*). The proposals have been assessed here depending on the degree of success that this feature is implanted with, or the available information about it. Table 5 portrays how fault tolerance is assessed for the proposals that have been regarded as relevant for this paper.

**Table 5:** Fault tolerance assessment.

Architecture Features	Grade Description
Grade: 5	A detailed description of how fault tolerance is present in the system is provided in the proposal. By this statement, it is meant that the procedures used to react against an unpleasant and unforeseeable event (e.g., system or hardware failures) are fully described. Implementation details, inner actions and performance results are also shown.
Grade: 4	A description on how fault tolerance is provided, although it is done from a more theoretical perspective and the data that have been obtained regarding its performance have been done so by using simulations rather than having a plethora of robots deployed and deliberately creating a disruption in the system to test it.
Grade: 3	Resilience mechanisms are described from a theoretical point of view rather than as something that has been implemented with a practical purpose. Data are shown about the procedures implemented by these mechanisms, but no information regarding tests is provided.
Grade: 2	Scarce data are offered regarding the resilience mechanisms of the proposal. There are just acknowledged in illustrations or in the available text of the proposals.
Grade: 1	Either no information is provided regarding resilience mechanisms or they are nonexistent in the proposal.

### 2.6. Scalability

By scalability it is meant the degree or the possibilities that an intermediation architecture as the ones that are described in the study can be extended to integrate a greater number of hardware entities. As it happened before, this feature is of remarkable importance as there are several cases where the number of underwater vehicles that has to be deployed will be higher than the one that was expected at first, and the intermediation architecture must guarantee that all of them can work easily in a coordinated manner. Table 6 shows how the evaluation of scalability has been done.

**Table 6:** Scalability assessment.

Architecture Features	Grade Description
Grade: 5	A specific mechanism is provided so that scalability is guaranteed in the system. The procedures that describe how a new hardware entity is included and how the system will behave to share information with it, as well as data being sent and received, are shown. Either there is a separated module to deal with this issue within the intermediation architecture or it is done by other parts of the system. Scalability has been tested in a real deployment.
Grade: 4	A mechanism to guarantee scalability is described thoroughly, often consisting of an autonomous, separated module. Implementation details, such as the programming language and the interaction with other parts of the system are shown. Information about its performance is present. However, it has only been tested in a simulation level, rather than with actual devices.
Grade: 3	Scalability is offered or even guaranteed to an extent, and diagrams or a description is offered to support this claim. A general overview of the procedures to guarantee scalability has been provided. Despite this, it is not clear how it is done, as there is no information about how scalability fares as a tested feature.
Grade: 2	Scarce information is provided with regard to the way to cope with scalability challenges in a very generic level. A mechanism or a procedure is described to an extent.
Grade: 1	No contingency plans are expected to be carried out in case scalability is required.

### 2.7. Context Awareness

Context awareness must be considered as a significant feature, since the context where the underwater vehicles are deployed might be of changing nature (water temperature, subsea substances composition, mission requirements, *etc.*). With context awareness enabled, better decisions can be taken regarding how to better tackle an issue (oil spills, seabed waste, *etc.*) so that the overall mission will be done in a more optimal way and fewer resources will be used. The evaluation of this feature has been described in Table 7.

**Table 7:** Context awareness assessment.

Architecture Features	Grade Description
Grade: 5	Context awareness is fully implemented as a mechanism to become aware of the conditions that surround the deployed piece of hardware. Its location in the middleware architecture is shown and the procedures to collect information from the context and use them are described. Implementation sketches and abundant details are provided. The module, facility or procedure that has been created to do so has been fully tested in a real deployment.
Grade: 4	Context awareness is provided as a service with a description profuse in details, and information on how it works is available as well. Procedures to collect information from the environment in order to take action based on it are formulated. However, its testing activities have been done with simulations in a controlled environment, so it is not clear how context awareness would be used in an actual deployment.
Grade: 3	Context awareness is provided as a black box where inputs and outputs are offered in a theoretical-only manner. A rough description on how it works is also provided without further details. Little information is offered regarding its performance.
Grade: 2	Scarce data is provided to take into account the context awareness feature. Diagrams and illustrations are available.
Grade: 1	No context awareness is provided, or it is done in a way that no effective information is provided.

### 2.8. Security

In a distributed system with devices deployed in a certain area security becomes one key feature to accomplish: if the information can be accessed by any agent not involved in the system the consequences could have a major impact; depending on the good or bad will of the alien participant, data could be altered, transferred to a hostile third party entity, erased or even replaced with something different and misleading. In the context of an underwater robotics deployment, information sent to the surface in order to have the responsible staff of the mission might be tarnished, resulting in the wrong decision making which would mislead the whole mission. Due to these reasons, security has to be born in mind when designing a middleware architecture for underwater robotics. The evaluation of this feature is depicted in Table 8.

**Table 8:** Security assessment.

Architecture Features	Grade Description
Grade: 5	Security is a fully fledged component in the intermediation architecture capable of covering a significant number of security functionalities (Authentication and Authorization, DDS based service, public key infrastructures), thus preventing any major security attacks to take place (Distributed Denial of Service, Man in the middle, *etc.*). The list of attacks that could be prevented is provided along with corresponding methodologies. Implementation and test details are provided in a profuse manner.
Grade: 4	Security is provided as a component or a module capable of preventing major security attacks to happen. Information about their inner performance is provided. Testing has been done with simulated environments rather than with actual underwarter vehicles.
Grade: 3	Security appears in the proposal, but it is either a scarcely described module or there are security functionalities in the proposal that are not explained in detail. Few data about its testing and performance are provided.
Grade: 2	Little information is provided about the security feature. Illustrations and/or diagrams have been provided to describe the security components of the proposal.
Grade: 1	No security is provided in the proposal, or there is not enough information to figure out its minimal performance.

### 2.9. Real Time

It is expected that a system as the one that is described here will be prone to using data transfers in real time. That is to say, there will be some pieces of information that will be required to be transferred as soon as they are retrieved from the context where the underwater robots are located in. In that way, decisions can be made at the center of the operations at a very fast pace. A common concern about this feature in underwater environments is that the bandwidth available for data transfers is rather narrow (usually around tens of kilobits), so data that are going to be sent in real time must be carefully planned. Table 9 summarizes the assessment that has been conceived for this feature.

**Table 9:** Real time assessment.

Architecture Features	Grade Description
Grade: 5	Real time functionalities are provided either as a set of methods or a module capable of sending and receiving information immediately to the upper levels of the middleware architecture without a significant delay. Information, such as code or diagrams, is provided. Implementations or tests in actual vehicles have been carried out with success.
Grade: 4	Real time functionalities are provided and a description on how they are tackled has been provided. Although there is plenty of information available, no code has been placed. Real time capabilities have been implemented and tested in a simulated environment.
Grade: 3	Real time features have been enabled in the proposal but there is little information about how data are transmitted from the lower levels of the architecture to the upper ones without significant delays.
Grade: 2	Very little data is provided about how real time capabilities are provided in the proposal, such as diagrams or a black box.
Grade: 1	No real-time capabilities have been conceived for the proposal or no information about them can be inferred.

### 2.10. Information Availability

When the proposal is a project by itself, or is included as a part of a larger project, the ability to ease the progress of similar solutions by means of the publication of information about them is also a quite compelling characteristic, as it will provide a starting ground for other projects while will still receive credits for it. The more information available (code repositories like Github, public deliverables, *etc.*) the easier it will be to get a grasp on the proposal by the community of developers. Besides, the open access to source code can enable achieving lots of valuable feedback, refinements or updates from the community. This feature has been assessed as described in Table 10.

**Table 10:** Information availability assessment.

Architecture Features	Grade Description
Grade: 5	A complete set of deliverables describing the steps and the overall proposal is available online and they can be downloaded. Among some other features, open source tools have been used and the resulting code can be downloaded from an online repository. The description of the proposal is an accurate depiction of what has been included in the available resources.
Grade: 4	Deliverables and code are available online in an open source manner, but the information provided by the deliverables is either too generic, or too cryptic. Code is of good quality and has been provided with comments that make it easy to understand and use.
Grade: 3	Deliverables are available to understand the scope and the aim of the proposal, but no code is available to download or share.
Grade: 2	No code is available in an online repository, or the proposal has been done using proprietary solutions that cannot be easily shared or ported.
Grade: 1	No resources that help have a better grasp of the proposal are available.

## 3. Description of the Proposals

A list of well-selected underwater intermediation architectures is provided in this section with brief introductions and comprehensive analyses based on the set of technical features that was introduced in the previous section. This list presents a holistic view on the newest research status of underwater robotics middleware, varying from the most widely used robotics middleware to novel proposals extracted from finished or ongoing projects.

### 3.1. Robot Operating System (ROS)

Robot Operating System (ROS) [20] is regarded as the most popular robotics middleware. It was firstly built in the STAIR (STanford Artificial Intelligence Robot) project with the involvement of Stanford University (Stanford, CA, USA) and Willow Garage (Menlo Park, CA, USA). It is designed as a very general-purpose middleware providing a set of facilities: publish/subscribe anonymous message passing, recording and playback of messages, request/response remote procedure calls and distributed parameters system. Apart from these components for general usage, additional robot-specific libraries and tools are offered for freeing users from developing applications from scratch. The exhaustive list of robot-specific features consists of standard message definitions for robots, robot geometry library, robot description language, preemptable remote procedure calls, diagnostics, pose estimation, localization, mapping, and navigation. All items of a robot system should register in a central server which is called ROS node in this case. Connections among nodes in ROS are established automatically in two manners. One method is passing messages through publish/subscribe model. Those messages do not need to be organized on the basis of a specific programming language. A node sends a message, which is simply a string, by publishing it to a given topic. Other nodes which are interested in a certain kind of data can get the notification of updates of this data by means of subscribing to this corresponding topic. The other method is using services with a request/reply framework.

A notable point regarding ROS is that it is an open source robot operating system and therefore it is free to be downloaded from its official website. ROS could be treated as a good foundation for developers to build more complex middleware solutions fitting specific requirements. Additionally, the community behind ROS is able to provide support and possibly open up the potential for more systematic solutions.

ROS needs more efforts (e.g., refinement, complexity, and adaptation *etc.*) to be upgraded and fine-grained so as to suit a particular usage. It is interesting to note that ROS has been chosen as a base and added with more functional modules to be a better match in many projects, such as Semantic Object Maps [25], Remote education [26] and Home service robot [27]. The reasons behind the wide adoption of ROS in such a variety of projects are manifold including ease of use, efficiency and scalability. However, security is not considered in ROS which could be regarded as future work. The assessment of ROS can be viewed in Table 11.

**Table 11:** ROS assessment.

Characteristic	Mark	Explanation
Data heterogeneity management	4	Information/messages are standardized and described by a language-neutral interface definition language. An appropriate data management should be chosen and tested in real environments.
System distribution	5	ROS is conceived as a distributed middleware composed of different nodes (interchangeable with software module) at a fine-grained scale. The distribution has been proven in many real projects.
Semantic capabilities	1	No semantic capabilities are included or even mentioned in the design. But it could be possible to add this feature in the next version of ROS as it can evolve to meet more requirements.
System services	5	A variety of system services are provided with detailed descriptions and implementations, such as service registry, debugging and configuration *etc.*
Fault tolerance	4	A component called Diagnostics provides a standard way to produce, collect, and aggregate information regarding analysis, troubleshooting and logging of the inner operation in robots. With the diagnostic information, it is easy to know the state of robot and address the fault detected in it.
Scalability	3	It provisions sharable resources like cores, cache *etc.* for guaranteed use by a process. It could be scalable to deal with increasing components or entities connected, but it lacks practical proof.
Context awareness	1	Context awareness is not considered in ROS, thus, no methodology is provided to endow ROS with this feature.
Security	1	No security mechanisms are considered.
Real time	4	ROS real time tools are available. However, the current capability is limited to provide the realtime publisher. Resource code is open released with relevant specifications. This feature is tested in simulations.
Information availability	5	Open sources are enabled regarding source code and documentations. Lots of publications regarding applications using ROS are available. Besides, the ROS community behind can be very useful for providing supports regarding ROS developments.

### 3.2. Distributed Robot Monitoring System (Drums)

Distributed Robot Monitoring System, abbreviated as Drums [28], provides a lightweight distributed monitoring and debugging tool for robot systems. It is conceived as a complement to other robot middleware systems with the target of facilitating resources such as introspection, monitoring, and debugging. This work has been motivated by a significant lack of awareness of failures and glitches in the underlying robotic middleware systems. It had been taken for granted that an ever-growing number of robotic middleware proposals work towards achieving a higher abstraction of heterogeneous hardware and software components. However, it is argued by Monajjemi *et al.* [28] that the disadvantage of high-level abstraction is that failures and resource constraints in the underneath mechanisms are not apparent. To compensate this missing part in current robotic middleware architectures, Drums is proposed as an important component for testing, debugging and run-time quality-of-service monitoring which could reinforce the holistic performance of middleware. In general, Drums is able to provide four kinds of functionalities:

- Generating and monitoring the computation graph which models the state of different resources in the middleware.
- De-abstracting and de-multiplexing abstract services, interactions, and communication channels into atomic and simple processes.
- Dynamically monitoring and diagnosing failures.
- Aggregating collected data into a central time series database in a cost-efficient way.

**Figure 2:**
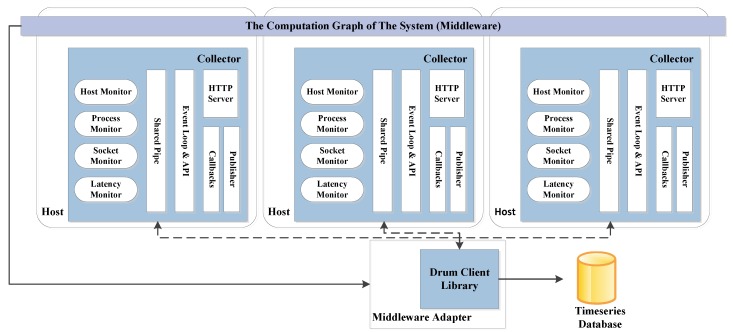
Overall architecture of Drums, as described in [28].

The realization of the aforementioned functionalities is conducted in two steps: creation of common representation model and monitoring the computation graph. More specifically, a generic model of robot systems regardless of specific adoption of middleware is defined so as to closely map native OS resources. Based on this model, information will be gathered to describe the specific state. The common model acts as a bridge loosely coupling the monitoring infrastructure and the middleware. Afterwards, the computation graph is constantly monitored over time so that inner statistics of resource usage can be obtained in a distributed manner. The conceptual architecture of Drums can be seen in Figure 2.

It can be inferred from this figure that Drums consists of three main modules: collector, client library and adaptor. Statistics collectors play a very crucial role in Drums providing the statistics from elements of the computation graph running on each host. Particularly, four kinds of monitors are implemented in the collectors, including process monitor (monitoring specific operating system processes), host monitor (collecting resources linked to utilization data about the host computer), socket monitor (capturing packets), and latency monitor (calculating latency between hosts and target tasks). Two different data retrieval methods are provided by the collectors: synchronous (polling with JavaScript Object Notation results returned) and asynchronous (publishing/subscription mechanism via ZeroMQ). The Drums client library is developed to provide an API to dispatch monitoring jobs to multiple collectors over the network. In addition to that the adaptor, in charge of monitoring the state of the middleware (hosts, processes and communication links), should be defined to fit into specific middleware platforms. A ROS-oriented adaptor has been developed in [28] by referring to directory services in ROS.

Drums provides an open access (see link: http://drums-project.github.io/) to its implementation and it has also demonstrated its usefulness in two tests: faulty router and excessive resource usage. It is true that Drums itself is not a complete middleware solution for robotic usages. Nevertheless, it could be regarded as a complement to be integrated with and work alongside other robot middleware to provide a transparent exposure of de-abstraction and de-multiplexing. In addition, it can be used as a low-cost data collection layer for fault detection and diagnosis systems. A future work direction can be developing a custom visualization and fault detectors for robotic middleware by use of Drums. Certainly, the possibility of taking advantage of Drums to detect excessive resource usage and anomalies and faults in the underwater swarms net can be explored. More detailed examination of Drums can be seen in Table 12.

**Table 12:** Drums assessment.

Characteristic	Mark	Explanation
Data heterogeneity management	4	Data is aggregated and organized into a central time series database.
System distribution	5	Different collectors can be mounted in different hardware using different adapters in a distributed manner.
Semantic capabilities	1	No semantic capabilities are included or even mentioned in the design.
System services	1	The middleware does not claim it could provide necessary system services, thus, insufficient information is described further.
Fault tolerance	5	Specific monitors are defined to dynamically monitor and diagnose failures. Information generated from the monitoring process is tested in two scenarios.
Scalability	1	No scalability mechanism is included.
Context awareness	2	Although this concept is not mentioned in the design, Drums can be regarded as using some kind of context aware because it can closely monitor changes of computation graph and provide diagnosis accordingly. However, the context awareness level is very limited.
Security	1	No information about security is included.
Real time	2	Very little information regarding this feature is provided. However, the proposal of latency monitor can be a useful hint.
Information availability	5	Open access to code is available with detailed tutorials. A few publications are also available for understanding this proposal.

### 3.3. CoHoN

A conceptual middleware, named CoHoN, was proposed in [29] to address the existing communication issues regarding heterogeneity inside a single robot and among multiple robots. CoHoN is aimed towards providing a decentralized, connection-based communication solution with very small message headers which goes beyond other existing robotic middleware solutions, such as ROS and Robotics Developer Studio (MRDS). A major concern in current robotic middleware research pointed out by Planthaber *et al.* [29] is that the majority of robotic middleware solutions introduce communication overhead while offering a considerable amount of beneficial capabilities. Keeping in mind a set of considerations, including usage simplicity, automatic setup, decentralization, Quality of Service (QoS), support for heterogeneous hardware, transparency, multi-robot support, dynamic data routing, and low overhead, a two-step approach including *setup* and *production* is proposed by CoHoN to facilitate the fulfillment of communication requirements from robotics. In the first phase (*setup)*, all possible routes will be searched within the network despite resulting in high network traffic. The reason behind the vast routing search is that the high network traffic does not affect the next phase production. *Production* phase can benefit from the setup phase with regards to attaching very small header information compared with traditional approaches.

**Figure 3:**
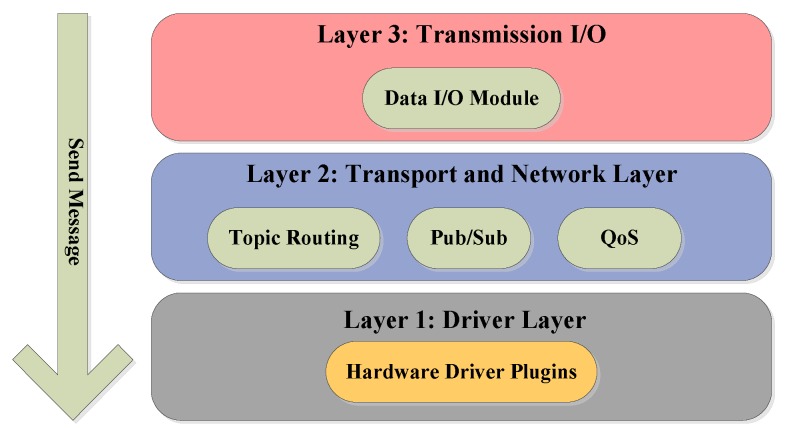
CoHoN architecture, as decribed in [29].

The CoHoN proposal is organized in a layered manner which is shown in Figure 3.

CoHoN consists of three layers: the driver layer, the transport and network layer, and the transmission layer. The driver layer is in charge of abstracting the various types of communication hardware in order to provide a common interface. This common interface enables communication between different types of hardware by means of setting parameters rather than rewriting programs. Additional transparent communications between network parts with different communication hardware are established in the transport layer. Multiple drivers can collaborate and act as relays to forward data towards different communication hardware types. In this way, multi-robot collaboration can be facilitated by subscribing data from another robot. Extra Data I/O functionalities, such as marshaling, encryption, redundant or parallel sending of data, are provided in the transmission layer using subscription process and topic routing.

The research on the CoHoN middleware still remains at the conceptual stage, although it is claimed that the routing principles used in CoHoN have already been simulated. Full implementation and further experiments should be continued and provided in the future. CoHoN cannot be treated as a well-designed proposal from the completeness aspect due to the fact that it lacks several functionalities necessary for a robotic middleware to operate in a satisfactory manner. In conclusion, the concept of CoHoN can be inspiring for renewing or even replacing communication procedures in current robotic middleware. An evaluation table (see Table 13) of CoHoN is provided.

**Table 13:** CoHoN assessment.

Characteristic	Mark	Explanation
Data heterogeneity management	1	Information regarding the specific method to manage data is non-existent.
System distribution	3	It is claimed the middleware is distributed and decentralized without descriptive explanations about the specific strategy applied.
Semantic capabilities	1	No semantic capabilities are included or even mentioned in the design.
System services	3	System services are fully described without complete implementation. They are pointed out as future work.
Fault tolerance	3	A resilience mechanism which can provide alternative routing in case of failures or disconnections is theoretically introduced. No practical tests are available.
Scalability	1	No scalability mechanism is included.
Context awareness	1	This concept is not taken into account.
Security	3	Security regarding encription is mentioned as an extra I/O functionality. However, it is not explained in detail.
Real time	3	Real time is mentioned to be guaranteed to some extent by a topic-based routing mechanism. It lacks detailed descriptions and further justifications.
Information availability	3	Publications and webpages are available to describle the purpose and funcationalities of this middleware. Code is not released as open source.

### 3.4. RAUVI Architecture

The Reconfigurable AUV for Intervention Missions (RAUVI) [30] project lasting 3 years from 2009 to 2012 asserts its interest in developing and improving the necessary technologies for autonomously performing an intervention mission in underwater environments. The revelation of the fact that a plethora of research activities have been conducted in the design of the architecture for individual autonomous vehicle or manipulator, thus resulting in a lack of work on the cooperation of both systems, motivated this project. Hence, how to facilitate the tight cooperation between a single autonomous vehicle and manipulator becomes the main focus of this project.

**Figure 4:**
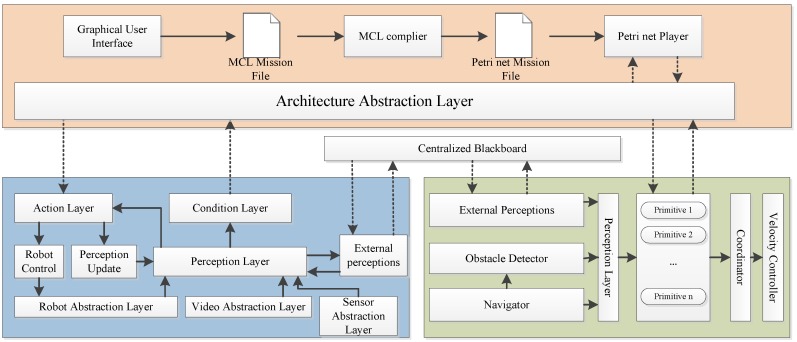
RAUVI architecture, as described in [31].

An integration architecture, which is named as Mission Control System (MCS), is proposed to interact between two control architectures initially designed and implemented for managing an AUV and an underwater manipulator, respectively, as depicted in Figure 4.

Figure 4 displays the whole architecture decomposition for the proposed RAUVI. The MCS is conceived to play an important role of interconnecting two separate architectures which are Manipulator architecture and Vehicle architecture. The joint architecture enforced by MCS allows a better plan and decision-making for intervention missions. As depicted from this figure, the MCS is composed of a set of building blocks including *Architecture Abstraction Layer*, *Graphical User Interface*, *MCL Mission File*, *MCL-Complier*, *Petri net Mission File* and *Petri net Player*. The main innovation of this MCS is the adoption of Petri net formalism [32] to define the task execution flow. In addition, Mission Control Language (MCL), as a friendly high-level language, is chosen to define Petri nets. Operators are able to provide extra information regarding localization of the target and specific grasping points for the knowledge of the AUV and manipulator. The essential approach to carry out the transmission of the specific mission plan and command is achieved by a clear interface based on actions and events.

The *Architecture Abstraction Layer (AAL)*, which resides between the MCS and the vehicle/manipulator architectures, is in charge of adapting these actions and events to the relevant instances of the target architecture. It is believed that different actions provided by the manipulator and primitives provided by the vehicle can be orchestrated so as to fulfill a complex mission within this proposed architecture. However, the cooperation is limited to a very low level which merely interweaves between a single AUV and a single manipulator. It cannot be treated as a full-fledged solution since it only takes two entities into account. The possibility of extending the usage of this proposed architecture to a larger area involving more vehicles and manipulators is worth to be explored. Besides, this proposed solution does not address the difficulty of data heterogeneity management. Semantic annotation capabilities are not included either. Many important features are missing in this proposal, such as guarantee of security for communications, data management, and capability of high-level inference. Further assessment of RAUVI can be seen in Table 14.

**Table 14:** RAUVI assessment.

Characteristic	Mark	Explanation
Data heterogeneity management	1	This feature is entirely missing in this proposal. No information is provided to introduce any data management mechanism.
System distribution	1	It is not conceived as a distributed solution.
Semantic capabilities	1	No semantic capabilities are included or even mentioned in the design.
System services	3	Capabilities, such as user interface, mission plan *etc.*, are provided with detailed descriptions, however, implementation lacks.
Fault tolerance	1	This feature is not taken into account in this proposal.
Scalability	1	No scalability mechanism is included.
Context awareness	1	It is a missing feature in this proposal.
Security	1	It is not taken into account in RAUVI.
Real time	2	Little information is provided to describe the specific strategy of supporting real time feature. It is only stated that tasks can execute in real time after a single Petri net generated.
Information availability	3	Publications and demos are available to introduce the idea of this middleware. Code is not released.

### 3.5. Pandora Architecture

**Figure 5:**
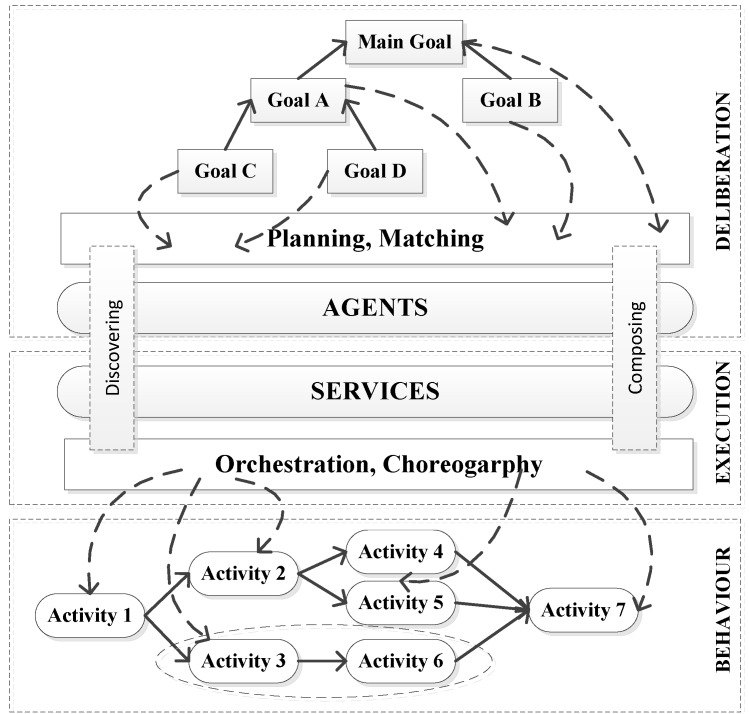
Conceptual view of the Pandora architecture, as described in [34].

The cognitive control architecture for autonomous marine vehicles [33] is conceptually structured in a layer-based manner, as shown in Figure 5. This proposed intelligent architecture aims to enable multiple marine vehicles to collaboratively perform missions with persistent autonomy. This architecture is built on the foundation of SOA (Service Oriented Architecture) where marine vehicles are treated as services. Different marine vehicles can integrate and collaborate with each other by connecting to the agent-based architecture. Each vehicle has various capabilities. All the functionalities will be encapsulated in different levels depending on the needs of specific mission. The process of encapsulation is called orchestration. More precisely, services are classified into four distinct types: Action service (atomic element of the service hierarchy), Task service (a set of Action services), Operation service (is composed of Task services), and Mission service (a group of Operation services). The component Choreography deals with the messages exchanged among services. Each marine vehicle will be deployed with one agent containing basic artificial intelligence blocks including mission planner and mission spooler (scheduler) *etc.*

**Figure 6:**
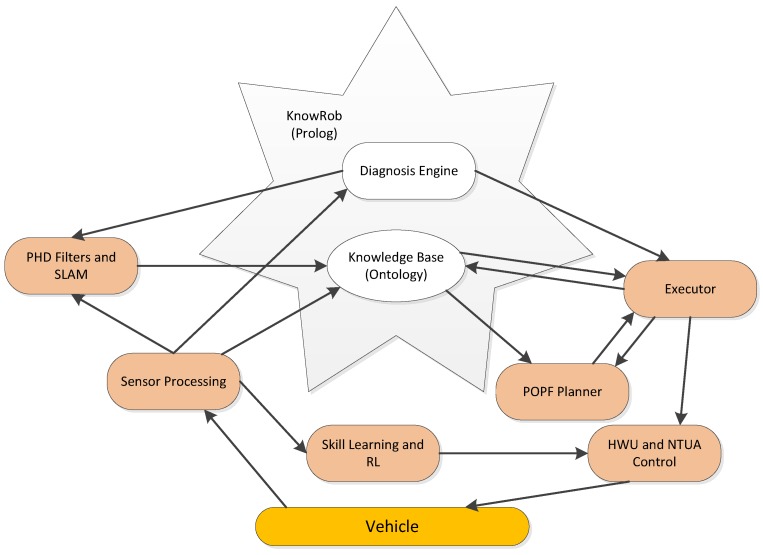
High-level architecture of KnowRob, as described in [37].

KnowRob [35,36,37] is chosen as the basis for implementing knowledge representation in the Pandora architecture. The high-level architecture integrating with KnowRob can be seen in Figure 6. KnowRob plays a central role of core knowledge repository in flexibly handling knowledge representation and offering interactions with other Pandora components via ROS connections. The general idea regarding data heterogeneity in Pandora architecture is that all data relevant to the Pandora project including vehicle’s state, operation and history will be stored in a set of ontologies which aim to describe the Pandora domain. All data formulated in the ontologies can be accessed by other modules for their own usages. *Executor*, as the key control module, will consult the knowledge base constantly for domain and mission data, encode that obtained information as PDDL (Planning Domain Definition Language), and finally invoke the *Planner*. Information can be directly queried by *Planner* for making planning.

The planning generated by the *Planner* will be passed back to the *Executor* to be executed using the robust control algorithms. It is worth noting that Pandora takes uncertainty inherent to the belief of world into account by introducing uncertainty related concepts into its knowledge model. Data sensed from the real environment will be fed into the ontology, either directly or filtered by *PHD/SLAM* [38,39] associated with an aggregated probabilistic estimate of certain state data. At the last step, the *Diagnosis Engine* will monitor the status of execution of the plan and give the feedback to the *Planner*. Results obtained from the whole procedure will be learned and integrated into the control modules by the *Skill Learning* module.

The proposed architecture is simulated on the basis of ROS, the results prove that the proposed solution can make it possible to achieve adaptive and reflective mission planning. However, it is detected that security is a missing part in this proposal. A significant discovery on this project is that it finally focuses more on development of new computational methods to make robots persistently autonomous, significantly reducing the frequency of assistance requests rather than enablement of collaboration of multiple vehicles as it was initially claimed. A summary of Pandora performance in terms of several criteria is shown in Table 15.

**Table 15:** Pandora assessment.

Characteristic	Mark	Explanation
Data heterogeneity management	5	The ontology-based approach to manage heterogeneity is described and tested in a controlled environment. Data storage is also enabled.
System distribution	4	Different agents consisting of necessary capabilities can be distributed in different vehicles or computation device. A simulated environment, rather than real test is described.
Semantic capabilities	4	Data semantics are included to infer more useful information which could be used for decision making purposes. A hierarchical ontology is created to describe the knowledge domain. The ontology is tested based on KnowRob at a simulation level.
System services	4	System services like service discovery, service composition, service choreography *etc.* are fully described and tested in a simulated environment.
Fault tolerance	4	Plan execution status is monitored by a diagnosis engine and passed to the planner to generate alternative plan. Actual deployment is missing.
Scalability	4	The adoption of KnowRob can handle an increasing amount of data. Besides, the concept “agent” can enable Pandora to accommodate more entities.
Context awareness	3	Although this concept is not specifically mentioned in this proposal it can be claimed as a service that it can hold. A mechanism on how context data is processed and deduced to produce more useful knowledge for enabling robots to adjust behaviors accordingly to the environment is introduced, which in fact exposes this potential capability.
Security	1	It is entirely missing in this middleware.
Real time	1	This feature is not addressed in this middleware.
Information availability	3	Publications, deliverables, and project webpages are available while code has not been released to the public.

### 3.6. TRIDENT Proposal

A multisensory control architecture, including a knowledge-based approach, to guarantee the suitable manipulation actions for enabling a multipurpose intervention system, is proposed in the TRIDENT project [40]. To this end, a cooperative team formed with an Autonomous Surface Craft (ASC) and an Intervention Autonomous Underwater Vehicle (I-AUV) is used to complete a series of sequential activities, including Survey (launching, survey, and recovery) and Intervention (launching, approaching, intervention, and recovery). The TRIDENT project (Marine Robots and Dexterous Manipulation for Enabling Autonomous Underwater Multipurpose Intervention Missions) is claimed to go beyond present-day methods typically based on manned and/or purposed-built systems by advocating the usage of this proposed architecture. It could offer intervention tasks for diverse potential applications like underwater archaeology, oceanography and offshore industries. TRIDENT project highlights its study on intelligent control architecture to provide an embedded semantic knowledge representation framework and high-level reasoning capability required to enable a high degree of autonomy and on-board mission decision making.

**Figure 7:**
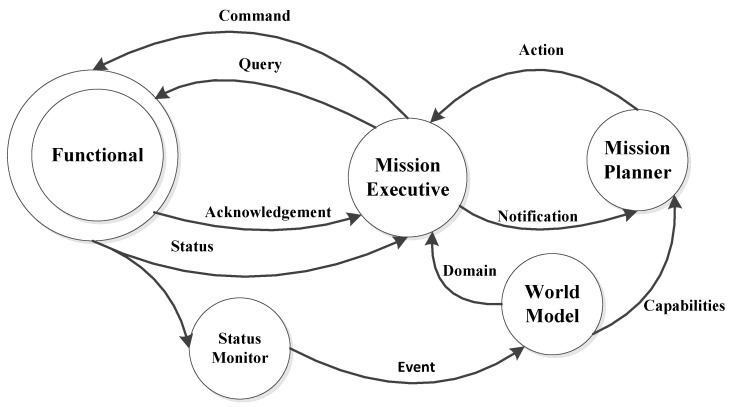
Semantic knowledge-based framework in Trident, as described in [41].

The semantic world model framework proposed in [41] is conceived to provide a capable and holistic system bent on integrating a set of autonomous underwater robots, in which semantic interoperability is achieved among various information sources. This semantic framework embedded in robot architecture is believed to enhance interoperability, independence of operation, mission flexibility, robustness, and autonomy. The whole architecture can be seen in Figure 7.

**Figure 8:**
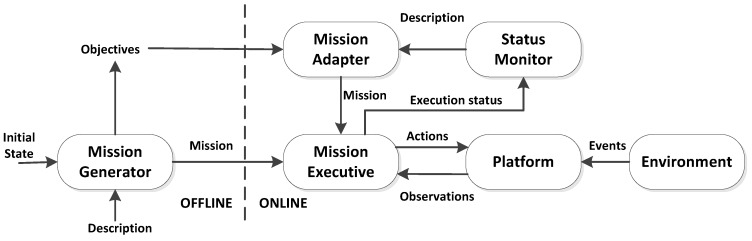
OODA loop for processing data, as described in [41].

The decision making process heading for the realization of situation awareness within this framework is based on OODA (Observe, Oriented, Decision, and Act) as Figure 8 shows. The objective of situation awareness is making the vehicle to autonomously understand the *big picture*. This *picture* is outlined by the combination of experience achieved from previous missions (orientation) and the information obtained from the sensors while on mission (observation).

**Figure 9:**
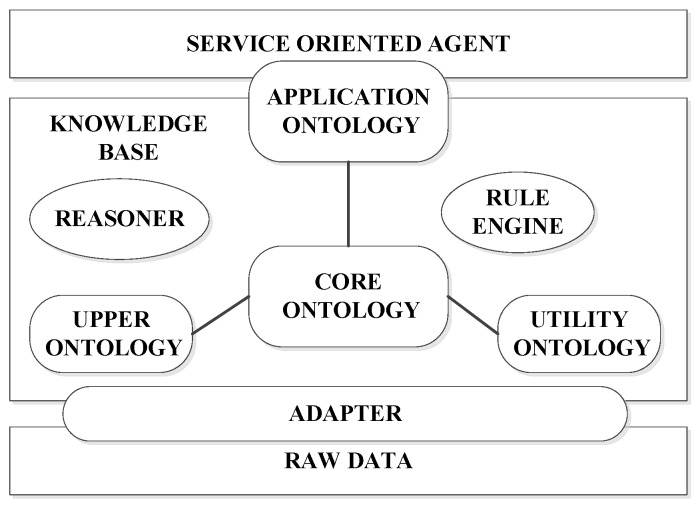
Situation awareness concept representation, instance generation and handling, as described in [41].

A hierarchical ontology model (depicted in Figure 9) plays a crucial role in enabling situation awareness by laying the knowledge representation foundation and guiding the decision making process.

Different ontologies are proposed at both core-oriented and application-specific levels. For example, a set of application ontology, which provides an underlying formal model for tools integrating source data and performing a variety of extended functions, includes status monitor application ontology and mission planning ontology. Core ontology includes several concepts like Platform, Payload, Module, Sensor, and Driver *etc.* Ontologies are utilized to represent the raw data obtained from robots and finally they are available for high-level decision-making agents.

The proposed architecture has been implemented and tested in real harbor experiments (Roses, Spain, October 2011), the results have shown that the cooperation between ASC and I-AUV is accomplished with the aid of status monitoring and mission planner. The initial design of this cooperation architecture is set as two entities involved which results in a limit of its application attempting to obtain cooperation in a bigger group of vehicles. Security, as an important factor, is not considered throughout this project. Future efforts can be put to apply the approach to more complex scenarios involving more agents and also address the acoustic communication limitations associated to the underwater environment. Trident assessment can be seen in Table 16.

**Table 16:** Trident assessment.

Characteristic	Mark	Explanation
Data heterogeneity management	5	Data heterogeneity is abstracted within a hierarchical ontology and tested in real harbor environments.
System distribution	4	It is conceived in a distributed fashion and prototyped in real scenarios. Details about maintenance tasks are not present.
Semantic capabilities	5	Data semantics are exposed to support the reasoning procedure. This feature has been proven in real scenarios.
System services	4	Various system services are offered and tested like registry and reasoning *etc.*
Fault tolerance	5	The status monitor keeps a close eye on how mission is executing and provides failure information for planner to deal with unexpected failures or disconnections. It is claimed that it has been shown in the harbor experiment.
Scalability	2	Scarce information is provided to justify the scalability issue. From the data management point of view, it is scalable to manage increasing data.
Context awareness	5	Context awareness is achieved following an OODA loop. Context data is obtained and processed to a great extent with the idea of generating useful information for robots to understand the underwater environment. A descriptive logic reasoning mechanism is applied. It is tested in real environments.
Security	1	Security is not considered in this middleware.
Real time	1	No information regarding real time is provided
Information availability	3	Publications and project webpages are availble to show this middleware design details. However, source code is not available.

### 3.7. Sunrise Proposal

An innovative concept of “the Internet of Underwater things”, firstly proposed by the European FP 7 Sunrise project [42], aims to bring about a revolution in current underwater communications. By the creation of an underwater “Internet of Things”, a group of underwater robots can interact with each other and work together. Thus, a maximum amount of collaboration between multiple robots can be achieved by means of good communications and data exchange between robots and computers via network, regardless of rapid changing environments and challenges to data transmission.

**Figure 10:**
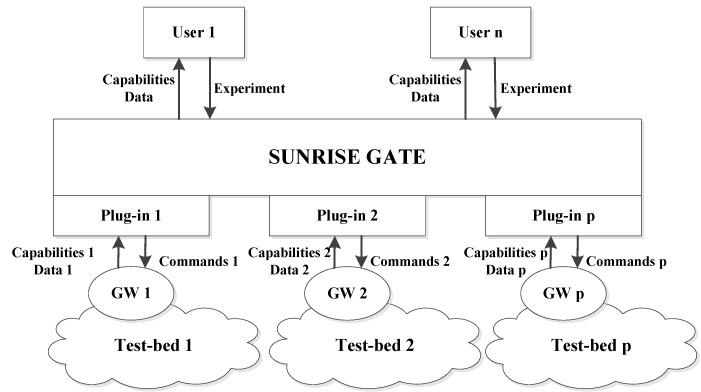
Conceptual schema of the SUNRISE federation architecture, as described in [43].

The underlying solution to infiltrate a connected “Internet” in the underwater environments is creating multi-vendor platforms that allow different types of devices to establish self-organization and task execution accurately, efficiently, reliably, and collaboratively. Thus, a federation architecture in Figure 10 is presented in this project.

The proposed federation architecture leads to a new paradigm in which seamless connectivity and monitoring between different experimental infrastructures are enabled. Taking a deep look into the architecture, it could be found that the SUNRISE GATE is actually acting as an intermediation layer which builds a bridge between various marine environments and diverse classes of added-value applications. Via this intermediated interface, users are able to run experiments and access data offered by different testbeds of the SUNRISE federation in a unified manner.

**Figure 11:**
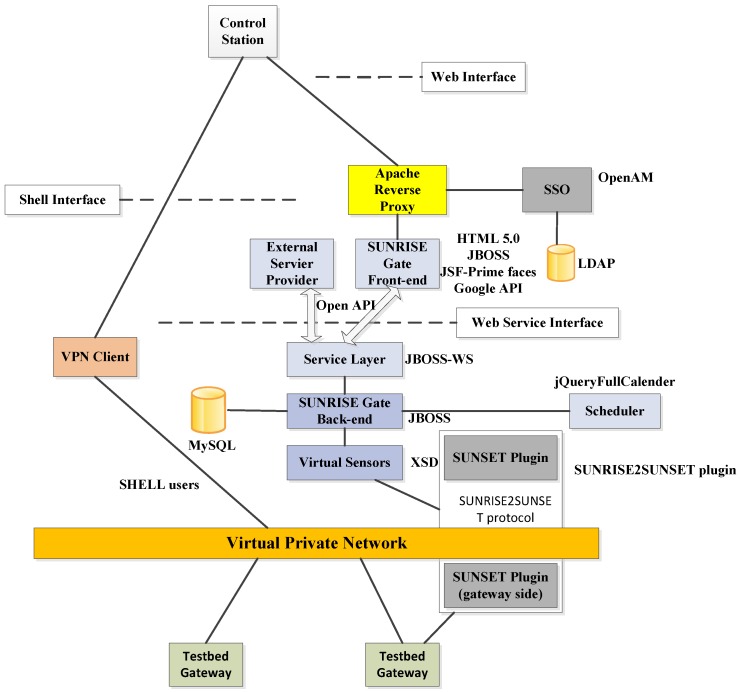
Breakdown of SUNRISE GATE, as described in [43].

As depicted in Figure 11, the general schema of the SUNRISE federation architecture could be summarized into 3 steps: (1) different assets involved in testbeds send data obtained from marine environments to gateways (GWs); (2) The GWs interacts with GATE by means of XML-based messages to register and upload information requested for the testbed. To do so, a set of plug-ins tailored to different testbeds is included to manage the communication and interaction between GWs and GATE; (3) GATE provides different means for users to develop applications which can manipulate and communicate with heterogeneous entities by exchanging commands and results.

A fine-grained breakdown of SUNRISE GATE can be viewed in Figure 11. GATE offers two different *Web interfaces* in a very friendly manner, namely *Web GUIs* for non-expert users with limited ICT knowledge and *Web Services* for ICT expert users. Through these interfaces, users are able to have a global view of data and services enabled in different testbeds. GATE provides a very good knowledge foundation for users to better design applications. What is more, in order to achieve a larger reachability for low-level functionalities offered by devices in the testbeds, a direct access through a suitable Operating System shell is created and available for users. It is worth noting that different security schemes are also introduced into GATE. More specifically, two modules including Single Sign On (SSO) and Lightweight Directory Access Protocol (LDAP) are inserted into this architecture to guarantee the authentication, authorization and accounting in GATE. It is stated that resources are managed in a hierarchical tree, which are federation, testbeds, devices, and network node in a top-down sequence. Data obtained from heterogeneous resources are formatted in a unified manner and stored in a MySQL database. However, the heterogeneity is only transparent and managed by plug-ins in a very low level where semantic annotations are missing. Future works could introduce semantics to endow heterogeneous data with a unified formalization and semantic meanings as well. Three testbeds like LOON, Porto Testbed and Buffalo have been deployed to test the performance of the proposed SUNRISE architecture. It is claimed that all testbed components have been extensively tested, validated and also refined. It proves that the SUNRISE architecture is able to support and integrate different testbeds in which diverse assets are involved.

Considering it is still an on-going project which needs a long way to live up to its promise of enabling a diversity of activities, further works should be continued to complete the design and implement it in more real usages. A holistic view of features provided by Sunrise can be seen in Table 17.

**Table 17:** Sunrise assessment.

Characteristic	Mark	Explanation
Data heterogeneity management	4	Various data are formatted and stored in a relational database, namely, MySQL.
System distribution	5	It is theoretically conceived in a distributed fashion and prototyped in real testbeds.
Semantic capabilities	1	This feature is missing in this proposal.
System services	5	A set of system services like application connection are provided. Particularly, registry is done by parsing xml-based messages. Security is ensured by SSO and LDAP. All these services are implemented and tested in real testbeds.
Fault tolerance	3	The status monitor keeps a close eye on how mission is executing and provides failure information for planner to deal with unexpected failures or disconnections. Proof of concept is missing.
Scalability	2	No detailed information is provided to justify the scalability issue. However, the concept “Internet of Underwater Things” in this proposal reveals possibilities to be scalable to connect more entities, though further details should be provided.
Context awareness	1	This feature is not mentioned in this proposal as well as relevant descriptions.
Security	5	Two modules are provided to guarantee authentication, authorization and accounting. This security mechanism is validated in real underwater environments.
Real time	4	Real time is included by the adoption of Sunset gateway. Details about the gateway are available.However, code is not available.
Information availability	3	Publications and project webpages are available. Source code keeps unreleased.

### 3.8. CoCoRo

The CoCoRo project [44] aims at creating an autonomous swarm of interacting and cognitive underwater vehicles. The swarm of vehicles is capable of conducting very specific tasks, such as ecological monitoring, searching, maintaining, exploring and harvesting resources. It draws inspiration from the nature so as to guide the behavior of individual vehicle to maximize their performance. Bio-inspired algorithms are widely applied in two kinds of usages: generating cognition and planning collective movement. Classic algorithms used in this middleware are Social insect trophallaxis [45], Social insect communication [46], Slime mold [47], ANN [48], Bird movement [49], and Fish school behavior [50]. The CoCoRo system could integrate several subsystems: a “floating base station” with the capability of feeding global information (e.g., GPS) into the system, a self-aware “ground swarm” performing the focal tasks and a “relay swarm” bridging the communication between these two subsystems.

**Figure 12:**
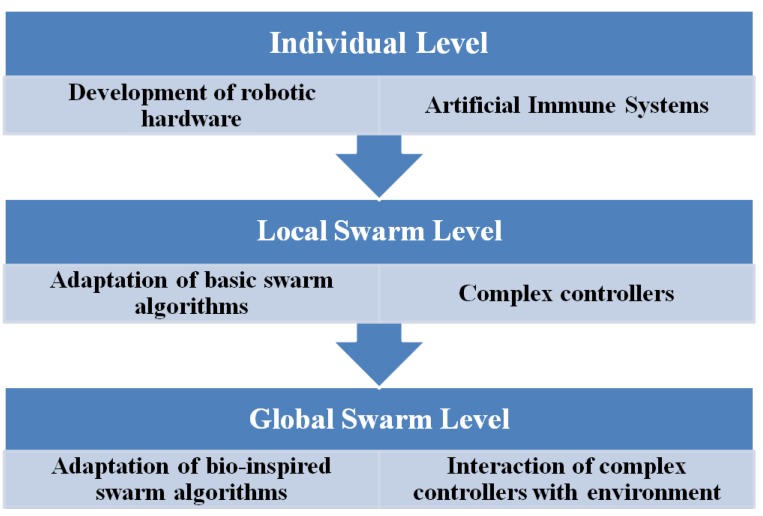
The whole picture of CoCoRo conceptualization, as described in [51].

The focus of this project is not put on the development of hardware. Instead, it lays its interests in the promotion of the application of bio-inspired algorithms into the underwater environment with the aid of appropriate hardware. Awareness of the surrounding can be achieved in a hierarchical manner, ranging from an individual level to a swarm level. As shown in Figure 12, individual AUVs can be aware of the environment through their own sensing devices. Further, short-range communication could be useful for enabling the awareness among groups of AUVs. The global awareness can be reached by introducing multiple emergent techniques, such as distributed memory, distributed map, distributed computing and advanced swarm algorithms.

In general, the CoCoRo system is structured as a decentralized and self-organized manner. A Unified Modelling Language (UML)-based framework is proposed in this project to capture and communicate the complex system. The UML model is able to detail specific entities, interactions and behaviors observed in the real world. However, the UML model lacks formal semantics and data sharing in spite of the profuse offer of well-explained diagrams.

Furthermore, CoCoRo does not provide a full-fledged middleware solution for the cognition and collective movement of swarms of AUVs. Nevertheless, CoCoRo’s novel bio-inspired algorithms could cast a new light into the expansion of the current state of the art in swarm intelligence and cognition to real cognitive robotic underwater swarms. It could be possible to abstract these control algorithms as building blocks to be embodied in other middleware solutions. In fact, a new project SubCULTtron [52] which is a follow up project designed after CoCoRo, will run until March 2019 with the aim of developing the largest underwater net that collects, coordinates and communicates data autonomously. The assessment of CoCoRo is demonstrated in Table 18.

**Table 18:** CoCoRo assessment.

Characteristic	Mark	Explanation
Data heterogeneity management	3	UML is chosen as the modelling method to formalize data needing more explanations about the specific procedure.
System distribution	3	System decentralization is claimed at a conceptual level without further proof.
Semantic capabilities	1	Semantic capabilities are unavailable.
System services	2	Limited services like individual cognition, swarms planning are introduced. No more details are provided.
Fault tolerance	3	Fault detection is not available. However, it could be possible to employ different bio-inspired algorithms to generate alternative plans in the presence of failures.
Scalability	3	It is not clearly mentioned or tested in this proposal. However, judging from the theoretical analysis in this proposal, it could be possible for this middleware to accommodate to a bigger group of vehicles.
Context awareness	4	Awareness of the surrounding is achieved in a hierarchical manner, varying from an individual level to a swarm level. It is tested in a simulated environment.
Security	1	Security is not considered in CoCoRo.
Real time	1	Real time is a missing feature in CoCoRo.
Information availability	3	Publications and project webpages are available. Source code is not provided.

### 3.9. T-REX

**Figure 13:**
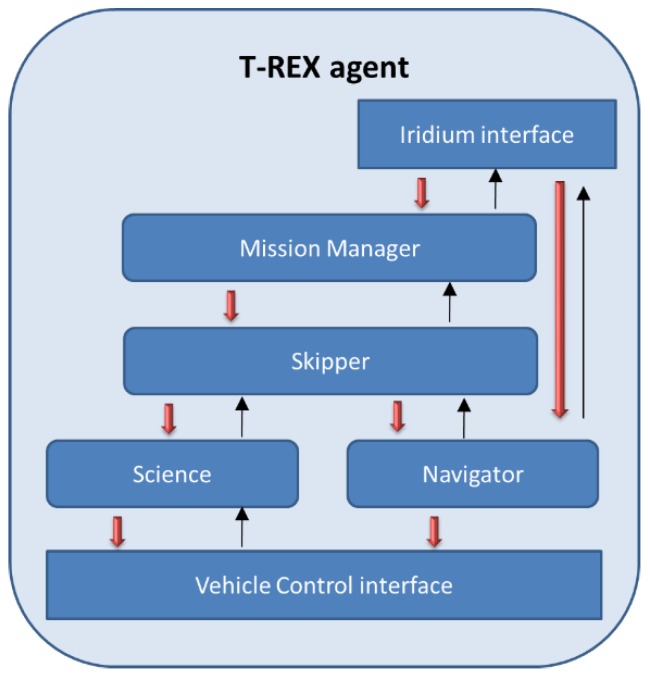
T-REX architecture, as described in [53].

The T-REX proposal [53] has been developed by the Monterey Bay Aquarium Research Institute (MBARI) as a way to have a software architecture capable of making decisions when underwater vehicles are confronted with changing conditions, such as water temperature or suspended particles. The authors claim that this architecture is based on the paradigm of *sense-plan-act*, as the vehicles that are deployed in a mission are able to sense the environment, make plans to successfully carry out a mission, and act according to the plan that has been established before. One key feature of this proposal is that missions can be replanned if it is required: should there be a sudden change in a variable that may have an impact in the mission, the latter will be replanned by one of the software modules able to react to short-term changes (called *reactors*). The architecture consists of several of these modules: two interface reactors (Iridium and Vehicle Control) use to interact with elements outside the software architecture, a mission manager for planning mission goals, and three more modules (skipper, science and navigator) used for low-level functional components of the vehicle. Figure 13 shows the overall structure of the proposal.

In this proposal, robustness is also guaranteed to an extent: the chain of reactor modules that is established guarantees that there will be an effort done to solve issues within an individual vehicle. Furthermore, it has been tested in actual missions, such as mapping, sampling of Intermediate Nepheloid Layers (INLs, suspended sedimentary particles lifted from the seabed to the sea surface) and analyzing temperature gradients in the sea.

Overall, the proposal proves that software architectures for underwater robotics are feasible in real missions; additionally, it is also proven how they can be capable of having a significant degree of autonomy and intelligence, as the architecture can adapt to changes done on the fly. Unfortunately, the proposal is also lacking a significant number of important features: it is not clear how distribution of information is done through all the deployed vehicles, and key characteristics as semantic capabilities or context awareness are missing from the proposal. Table 19 shows the assessment of T-REX.

**Table 19:** T-Rex assessment.

Characteristic	Mark	Explanation
Data heterogeneity management	3	Sample collection of different elements found in the sea and underwater mapping have been proven to be done in different missions. It is not clear if there is a software module devoted to sending and receiving data throughout all the deployed underwater robots, or how the proposal transfers information outside the vehicle.
System distribution	2	The proposal portrays the deployed robots as autonomous and capable of taking decisions by themselves. A description of a procedure to coordinate activities among several of them is not provided, nor are details regarding distribution given.
Semantic capabilities	1	No semantic capabilities are mentioned in the proposal.
System services	5	The proposal is capable of providing a wide range of services in missions, such as sampling, mapping and finding suspended particles in the sea. The most prominent of these features is the analysis of Intermediate Nepheloid Layers (INLs). Machine learning techniques are claimed to be used as well.
Fault tolerance	4	The system establishes a hierarchy, relying on a chain of reactors until a solution is found for the problem. Replanning a mission when unforeseen events take place can also be done. The usage of other underwater vehicles that might support a failing one is not explained.
Scalability	2	The autonomy of the underwater robots enables their extended usage, but it is not explained how they coordinate one with the other, or to what extent the chain of reactors can be extended.
Context awareness	2	It is mentioned that there is a reaction from the environment and actions can be taken regarding this reaction, but there is not a detailed explanation on how to do so, nor any diagram is provided.
Security	1	No security measures are mentioned in this proposal.
Real time	2	The real time used by the operating system installed in the robots is real-time based, so T-REX could be able to handle request of that nature. Yet it remains unconfirmed as far as the middleware architecture is concerned.
Information availability	2	Scientific publications are available online, as well as the initiative where it was tested [54]. Open source resources are claimed to be used. No code or deliverables have been found.

### 3.10. Huxley

**Figure 14:**
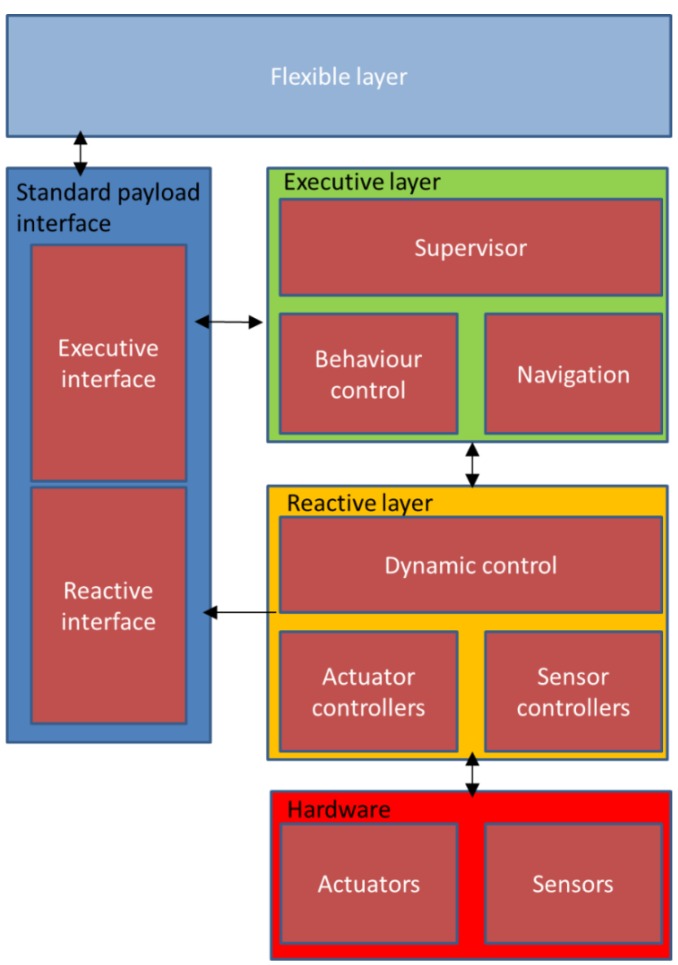
Huxley architecture, as described in [55].

The authors of the Huxley proposal [55] regard it as a production robot control architecture enabled to be adopted in a wide range of platforms, that is to say, underwater vehicles. Flexibility, understood as the capacity of using the proposal with a variety of platforms and payloads, is a feature greatly stressed in this architecture. Apart from that, the authors claim that there are four different features of major importance that have been taken into account: reliability (having common elements and an underlying infrastructure to deliver a core software system), extensibility (capability to add new capabilities and enhancements), maintainability (ability to keep the proposal usable as it increases its complexity) and testability (deploying the proposal across the supported platforms or vehicles). Figure 14 displays the overall structure of Huxley.

As can be seen from the Figure 14, there are several components in this architecture that are linked to reactive functionalities, just as it happened in the previously studied proposal. There is a flexible layer (which is used as the software payload), a standard payload interface (with the proper executive and reactive interfaces), an executive layer (used for supervision, navigation and behavior control) and a reactive layer (used for dynamic control and equipped with the suitable controllers for sensors and actuators) that is interchanging information with the installed hardware (actuators and sensors). The executive interface is used to send and receive data requests, whereas the reactive interface only receives information from the architecture rather than initiating any procedure. In addition to this, Huxley makes use of a publish-subscribe messaging protocol to transfer data named Stream-Oriented Messaging Architecture (SOMA), which is basically used to unify the former elements of the architecture and establish peer-to-peer connections between applications or processes.

According to the authors, Huxley has been tested in different robotic units and participated in several missions (see system services in Table 20). However, while the proposal has been quite used in actual deployments since 2005, it shows a low level of refinement, lacking several major features of latter developments, such as semantic capabilities or context awareness. In addition to that, it is not stated how all the elements of a deployment are communicating one with the other, or how system distribution is done. Table 20 shows the evaluation of the proposal.

**Table 20:** Huxley assessment.

Characteristic	Mark	Explanation
Data heterogeneity management	4	The proposal is used by several different underwater vehicles of different features, so data of different characteristics can be managed. There is no information regading interconnectivity among the different vehicles once they are deployed.
System distribution	1	No information about system distribution is provided in the proposal.
Semantic capabilities	1	No semantic capabilities are mentioned in the proposal.
System services	5	The proposal has been used for different tasks with a considerable degree of success; applications mentioned are mine-countermeasures or battlespace preparation. Interest is shown in expanding those activities to persistent surveillance and anti-submarine warfare (ASW).
Fault tolerance	2	Fault detection and recovery are mentioned as a feature tackled by the executive layer, but it is not described how it is provided.
Scalability	2	Scalability is mentioned as extensibility in the proposal, but little information is provided about it, and it seems not to imply scalability in the number of vehicles than can be used.
Context awareness	2	Context awareness is only hinted in the proposal, mentioned as a will to make observations of the world around the hardware entity and take actions within that framework.
Security	1	No security measures are mentioned in this proposal.
Real time	1	No real time features are mentioned in the proposal.
Information availability	2	Huxley runs in an open source Linux environment [56]. No code or extensive documentation (tutorials, deliverables, *etc.*) has been found online.

### 3.11. LCM

Lightweight Communications and Marshalling, abbreviated as LCM [57], provides a set of tools for message passing and data marshalling, simplifying the development of low-latency message passing system (especially, targeting at real time robotics applications). As modularity has become a fundamental design principle in most robotics middleware, it is convenient to map individual modules onto software processes which can be distributed in different physically separate computation devices. Thus, the issue of information exchange between different modules can be referred to the well-studied problem of interprocess communications. Aiming at tackling the information exchange between different modules within robotics middleware, LCM is designed as an “*a la carte*” communication system with focus on debugging, analysis, and deep inspection of messages passed between modules instead of being a complete operating environment.

**Figure 15:**
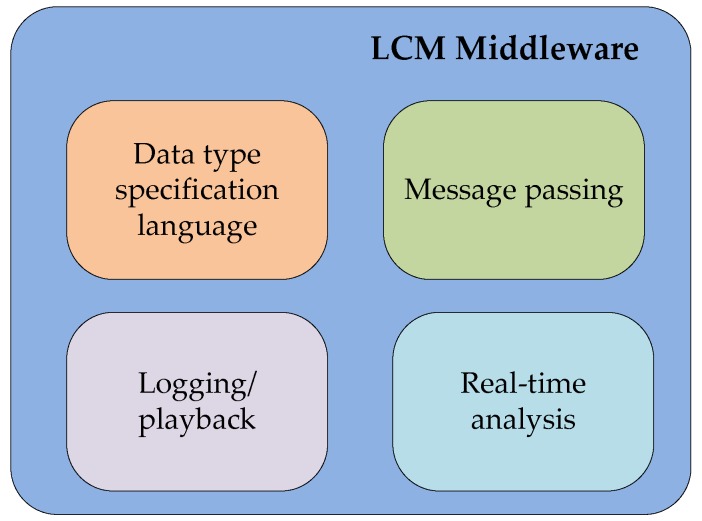
LCM middleware, as described in [58].

As shown in Figure 15, LCM is formed by four components: a data type specification language, a message passing system, logging/playback tools, and real-time analysis tools.

A platform- and language-independent type specification language, referring to the method and syntax for defining compound data types, is provided by LCM. This type specification language will be agreed among processes which wish to communicate and be used to formalize data that will be exchanged. The LCM type is strongly influenced by External Data Representation (XDR) [59] which is used by the Player middleware. However, some XDR features are not included in LCM type because of their rare use and implementation difficulty. In addition, some features and usability improvements over XDR are offered by LCM, such as allowance for declaration the length of a variable length array and support for namespaces. In general, the type specification language specifies the structure of a message, thus implicitly defining how the message is represented as a byte stream.

The procedure of encoding and decoding of structured data into an opaque binary stream that can be transmitted over a network is called marshalling and unmarshalling in LCM. LCM provides automatic generation functions for marshalling and unmarshalling of user-defined data type into specific data type oriented to specific operating system. By employing those functions, different modules can agree on exactly how to interpret contents of a message, thus reach the same understanding on a single message. Fingerprint, as a hash of the member variable names and types, is encapsulated in the type definition. When a message is received by a client, the fingerprint will be read to be checked if it matches the one expected by the LCM client. A type error will be detected and reported if they are not identical.

LCM implements a publish/subscribe model using UDP multicast as its underlying transport layer. LCM communications differ from typical publish-subscribe systems in eliminating the need for a central communications hub. It simply broadcasts all the messages to all clients which may result in a wasteful use of resources. For this reason, UDP multicast comes in handy as it provides standardized protocols and programming interfaces. Low latency is favored in LCM over guaranteed delivery semantics. The reasons are twofold. On the one hand, robotics systems have significant real-time constraints in which a lost packet is preferred to be dropped so as not to delay future messages. On the other hand, the loss of a message may not be the result of a transient network failure so unreliable yet low latency communication is preferred and adopted in LCM. Last but not least, LCM develops a number of logging, playback, and traffic inspection tools simplifying common development and debugging tasks. Developers can benefit from those tools with regard to rapidly and efficiently analyze the behavior and performance of LCM.

LCM has been deployed on a number of robotic systems on land, water, and air. Particularly, it has been verified by a number of AUVs at MIT (Cambridge, MA, USA), University of Michigan (Ann Arbor, MI, USA), and the Woods (Woods Hole, MA, USA). LCM experiments on AUVs regarding conducting tasks like underwater robotic mapping, cooperative multi-vehicle navigation and perception-driven control have been proven just as useful. LCM also provides open access (https://lcm-proj.github.io/) to its source code for use. In general, LCM can be regarded as an alternative for implementing interprocess communication (IPC) for real-time robotics in the marine environment. It could act as a well-designed low-latency communication infrastructure, by means of permitting message loss to minimize latency of new messages, which could be conceptualized as an independent communication module and integrated in other middleware architectures. The overall assessment of LCM is shown in Table 21.

**Table 21:** LCM assessment.

Characteristic	Mark	Explanation
Data heterogeneity management	4	Heterogeneous data are encoded and decoded (referring to as marshalling and unmarshalling) for exchange among different modules according to the LCM data type specification language. This mechanism has been tested as useful in real scenarios.
System distribution	5	It is a modular system which can be distributed in different physically separate computation devices. The distribution feature of LCM has been shown in a number of real robotics applications, such as land and underwater vehicles, autonomous indoor flight *etc.* Extensive information regarding implementation guide are presented in the LCM’s website.
Semantic capabilities	1	Semantic capabilities are not available in the LCM middleware.
System services	2	Communication related services, like marshalling, unmarshalling, debugging, and analysis are fully described, implemented, and tested. No other kinds of services (like service registry, decision making *etc.*) beyond this are provided.
Fault tolerance	1	This feature is not taken into account in this proposal. Thus, no fault tolerance scheme is mentioned.
Scalability	3	It is claimed that communications can be ensured in an increasing number of modules as UDP multicast is used to guarantee high bandwidth and scalability. Nevertheless, no specific module is designed to deal with this issue.
Context awareness	1	No information regarding the context awareness feature is provided in this proposal.
Security	1	No security mechanisms are mentioned in this middleware.
Real time	5	A module, called real time analysis, is devoted to enabling this feature. Details about the specific mechanism are provided. Besides, this feature has been tested in actual vehicles.
Information availability	5	Scientific papers and webpages are available to understand the internal composition of this middleware. Besides, source code regarding different platforms (Linux, OS X, Windows and any POSIX-1.2001 system) and different languages (including C, C++, C#, Java, Lua, Matlab and Python) is released.

### 3.12. MORPH

The proposal named as Marine robotic systems of self-organizing, logically linked physical nodes (MORPH) [60] provides a set of tools for the coordination and cooperation of AUVs to obtain the mapped layout of underwater structures. A common architecture has been defined to achieve this goal using a heterogeneous set of vehicles. The main idea is the concept of a virtual vehicle, called MORPH supra-vehicle, as the result of the composition of the capacities provided by different physical vehicles, called MORPH nodes. The typical configuration comprises the following vehicles:

- A Surface Support Vehicle (SSV).
- A Global Communication Vehicle (GCV), used to improve the navigation accuracy and relay communications.
- A Local Sonar Vehicle (LSV), equiped with one multibeam echosounder.
- A couple of camera carrying vehicles (C1V and C2V).

As this proposal is tightly coupled to the specific goal of mapping underwater structures, so the components, or modules, of the proposed architecture are focused on tasks related to the fulfillment of this goal.

**Figure 16:**
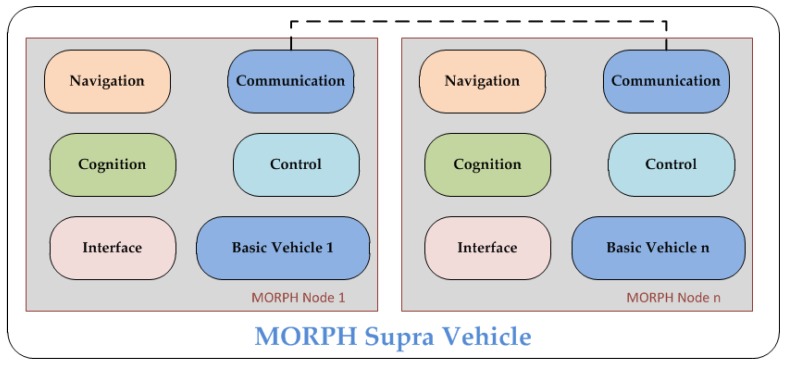
Architecture of a MORPH node and MORPH Supra Vehicle, as described in [60].

Figure 16 denotes the architecture proposed in MORPH. A MORPH supra-vehicle is composed of several MORPH nodes aimed to complete the mapping of an underwater structure. Each MORPH node is a real AUV capable of performing some specific tasks, while the MORPH supra-vehicle can be seen as a virtual vehicle as the result of the coordination of each node contributing to the supra-vehicle.

Each individual node consists of MORPH hardware and software modules that enable the participation in the supra-vehicle composition. MORPH software modules are linked to an intra-node communication bus. ROS is used as a communication middleware, although some vehicles in the MORPH project are also capable of using MOOS [61].

There is one main module providing the interface between the rest of the main MORPH modules and the existing vehicle hardware and software. This module has to be adapted to each specific vehicle. The remainder of modules provide functionalities for the generation and supervision of communications, relative navigation of the nodes with respect each other, cognition and control. The overall assessment of MORPH is shown in Table 22.

**Table 22:** MORPH assessment.

Characteristic	Mark	Explanation
Data heterogeneity management	2	From the diagrams and publications it seems clear that there must be some kind of data heterogeneity management, as the system provides coordination between heterogeneous vehicles. But there is no clear or relevant information regarding this topic.
System distribution	4	The system is distributed using a common set of modules to provide a composed view of all the vehicles as one.
Semantic capabilities	1	There is no information about data semantics.
System services	3	Services have been defined and tested in real environments. However, they are tightly coupled to the specific goals of the project.
Fault tolerance	2	No specific information has been provided regarding fault tolerance. The description of the project and the architecture acknowledge that some resilience mechanisms should be implemented.
Scalability	3	The definition of the interface module provides the capacity of adding new hardware entities or vehicles to the system. The description of the interface module is lacking information.
Context awareness	3	Context awareness, as defined in this proposal, is provided by the cognition module. No details have been provided on the implementation and performance of this module.
Security	1	No information about security mechanisms is available.
Real time	3	There is no information related to real time data transfers.
Information availability	2	Source code and architecture details are not open. A collection of publications regarding algorithms developed to achieve the goals are available.

## 4. Main Issues and Challenges

After presenting and introducing each existing middleware proposal individually, it is worth making a thorough comparison of them so as to have a clearer understanding of the latest research status of underwater robotics middleware. A summary of presented middleware proposal is provided in Section 4.1. Based on the study, several open issues which remain as challenges in current underwater robotics middleware research are pointed out in Section 4.2.

### 4.1. Comparisons of the Proposals

In order to consider the overall assessment that has been done with all the proposals, Table 23 shows the scores that they have obtained, according to the criteria that were described in Section 2.

Table 23 shows the whole performance of the different presented middleware solutions in terms of the set of technical considerations, including data heterogeneity management, system distribution, semantic capabilities, system services, fault tolerance, scalability, context awareness, security, real time, and information availability. As shown in this table, different middleware proposals differ from each other to a great extent. Taking the total score as the holistic performance indicator, their performances greatly vary from the lowest (15 points obtained by RAUVI) to the highest (35 points obtained by Trident). However, none of them are fully versatile with regards to having complete implementations of all considered features.

Due to major differences in original interests of the researched works and the specific approaches applied, these studied middleware proposals result in a wide diversity of matches within underwater robotics usages. According to the relevance to the scope of interest (namely, underwater environments) in this paper, RAUVI, Pandora, Trident, Sunrise, CoCoRo, T-REX, Huxley, LCM and MORPH outperform the other ones (including ROS, Drums, and CoHoN) because they are keeping the specific domain as their initial design goals. On the contrary, ROS, Drums and CoHoN are designed as basic, yet generic platforms conceived to support robotic research regardless of the specific domains where they are applied, which will require more extensions and adaptations when attempting to fit in underwater environments. As far as completeness is concerned, Drums, CoHoN, CoCoRo, LCM seem barely satisfactory to be labeled as fundamental middleware since they only cover functionalities limited to tackle a single objective. For example, Drums, designed to provide distributed monitoring and debugging tools, is regarded as a complement for other robotic middleware systems. Similarly, communication heterogeneity is the only issue considered in CoHoN. CoCoRo makes a considerable amount of contributions to improve swarm intelligence by applying bio-inspired algorithms, but significant features (e.g., data heterogeneity management, semantic capabilities, and system services *etc.*) are neglected. Guaranteeing low-latency communications in real-time constrained systems (particularly, focusing on robotics system) is the primary focus of LCM. Nevertheless, the possibilities of integrating Drums, CoHoN, CoCoRo or LCM into other middleware or replacing corresponding parts in other middleware can be further explored so as to fully make use of their innovations.

**Table 23:** Comparison of reviewed middleware proposals.

Middleware Proposals	Data Heterogeneity Management	System Distribution	Semantic Capabilities	System Services	Fault Tolerance	Scalability	Context Awareness	Security	Real Time	Information Availability	Total Score
ROS	4/5	5/5	1/5	5/5	4/5	3/5	1/5	1/5	4/5	5/5	33
Drums	4/5	5/5	1/5	1/5	5/5	1/5	2/5	1/5	2/5	5/5	27
CoHoN	1/5	3/5	1/5	3/5	3/5	1/5	1/5	3/5	3/5	3/5	22
RAUVI	1/5	1/5	1/5	3/5	1/5	1/5	1/5	1/5	2/5	3/5	15
Pandora	5/5	4/5	4/5	4/5	4/5	4/5	3/5	1/5	1/5	3/5	33
Trident	5/5	4/5	5/5	4/5	5/5	2/5	5/5	1/5	1/5	3/5	35
Sunrise	4/5	5/5	1/5	5/5	3/5	2/5	1/5	5/5	4/5	3/5	33
CoCoRo	3/5	3/5	1/5	2/5	3/5	3/5	4/5	1/5	1/5	3/5	24
T-REX	3/5	2/5	1/5	5/5	4/5	2/5	2/5	1/5	2/5	2/5	24
Huxley	4/5	1/5	1/5	5/5	2/5	2/5	2/5	1/5	1/5	2/5	21
LCM	4/5	5/5	1/5	2/5	1/5	3/5	1/5	1/5	5/5	5/5	28
MORPH	2/5	4/5	1/5	3/5	2/5	3/5	3/5	1/5	3/5	2/5	24

ROS, Pandora, Trident, and Sunrise (which are ROS-based implementations) are more competitive compared with the other middleware solutions with regards to the quantity and quality of services they provide. In other words, they cover more possibilities when trying to meet various requirements from the underwater environments by offering several system-level services. It is worth noting that most of the presented middleware architectures do not take security into account, except Sunrise and CoHoN. More specifically, Sunrise is the only one providing a complete mechanism to tackle security issues while CoHoN merely mentions that it could enforce encryption as one of extra I/O functionalities without elaborate clarification.

Organizing structures in a distributed manner has been taken into account as a common basis adopted by the majority of presented middleware solutions, except RAUVI, T-REX, and Huxley. Data heterogeneity is not well considered in four proposals, including CoHoN, RAUVI, CoCoRo, and T-REX. Trident and CoCoRo show their cutting-edge achievements regarding context awareness while research efforts in other proposals still remain at a nascent stage. Due to the dramatic increasing need of integrating and coordinating an ever-growing number of vehicles in underwater environments, scalability should be better conceived in the existing proposals. Introducing semantic capabilities is also worth being considered to reinforce existing middleware in terms of making better use of information. As far as fault tolerance is concerned, more improvements should be made in the presented proposals, especially in RAUVI and LCM which entirely lack this feature.

The capability of establishing real time data transfers is highly demanded in the underwater environments since it is beneficial to support fast and context aware response to the changing environments. With regard to this feature, LCM, Sunrise and ROS have shown better considerations over other middleware solutions.

Information availability should be considered in all these middleware as the availability of open source or commercial popularizing of enough significant importance to broadcast the software and advocate its usage. Due to limited information availability in some middleware solutions, such as T-REX, Huxley and MORPH, it is difficult for developers to have a good and thorough understanding of their essential design principles and further have a fair evaluation on them.

### 4.2. Open Issues

Based on the analyses on existing intermediation architectures, a set of challenges has been detected and presented as open issues to point out potential future improvements.

#### 4.2.1. Lack of Implementation Works

Due to the fact that the application of middleware in cooperative underwater robots is still in a quite emerging state and overall under-researched, the majority of work efforts are still staying on the conceptual stage. More specifically, many middleware solutions are proposed associated with theoretical analyses and design philosophy while proof of concept is missing. The research on those middleware architectures is far from complete implementation, let alone thorough validation and evaluation, e.g., Trident and Sunrise provided their first attempts to show the performance of their proposed solutions by making prototyping tests. Nevertheless, the implementation details still remain unclear. In addition to that, driving those middleware solutions to commercial usages also looks like a difficult task in the future.

#### 4.2.2. Unsatisfactory Performance

The performance of middleware architectures does not live up to what they are expected. Many middleware proposals are claimed by their designers to hold powerful capabilities which aim to tackle a lot of notorious difficulties, such as context awareness and interoperability. However, the real fact is the result lags behind the statement to a great extent. Those stated features are not even considered and covered during the implementation phase. In other cases, middleware implementations only reach partial requirements despite of using those approaches they proposed at the beginning.

#### 4.2.3. Security

Security is a constant hotspot that attracts a lot of attention and requires extra efforts working on it. However, security is a missing feature in the majority of current middleware proposals. The issue of security can be divided into several aspects considering the particular domain of interests, here which refers to underwater environments: (1) *Security imposed on data*. Data obtained from underwater environments should be protected from being interpolated or faked during the lifecycle. Relevant security mechanisms should be introduced into two sectors: communication and data storage. (2) *Restriction on user access*. Only authorized users can interact with middleware: send requests, issue a command and consult for information *etc.* Otherwise, robots could be dangerous by relying on commands sent from unauthorized users. (3) *A lightweight identification scheme* should be established between middleware and vehicles. Data exchanged from middleware and vehicles can be identified regarding authentication and integrity so that trustful communication can be built.

#### 4.2.4. Consideration for Low Capable Vehicles

The constraints for underwater vehicles when deploying middleware onboard should be born in mind. It will not be a problem to deploy the entire middleware components embedded in a self-constrained vehicle. However, when the middleware tries to facilitate the integration among a group or even a swarm of vehicles, vehicles with low capabilities should be considered because of low computational capability, bandwidth scarcity and insufficient memory, *etc.* The deployment of middleware architectures should be carefully designed: entirely onshore, or entirely onboard, or partially onboard, or distributed in different entities to meet individual needs.

#### 4.2.5. Low Context Awareness Level

Context awareness held by existing middleware solutions for underwater environments is very limited. Considering the uncertainty and ever-changing nature of underwater environments, it is needful to be aware of the surroundings so as to support the middleware with the capability of making corresponding adaptations. It would be nice to put more efforts in adding the concept of context awareness in existing middleware solutions. In order to correctly implement context awareness, context extraction and processing are time and resource consuming. There is always a trade-off between the amount and quality of the gathered information and the actual gain achieved from a more accurate context. Determining this balance is one of challenges for underwater robotics middleware. Policy constraints can be applied at early stages to reduce the wasted resources in context extraction.

#### 4.2.6. Consideration of Data Imperfection

The nature of data obtained from the underwater environment is usually flawed due to a lot of factors: imperfect instruments, noise, partial views, occlusions, harsh measurement conditions, *etc.* The imperfection of the obtained data imposes different kinds of uncertainty for data processing. On the one hand, data measured from the environments may not represent the real change of observed aspect. It could be uncertain with a certain degree of authenticity if compared to the original data, or repeated measurement values on a single phenomenon can be variable within a variance range. On the other hand, it is likely that ambiguity will exist at the population of individual data into the predefined ontology. There is no doubt that ontologies are becoming more popular in modelling information by means of middleware solutions. However, the majority of middleware solutions only make use of crisp ontologies which are unable to manage uncertainty and vagueness [62]. It is worth making efforts to manage uncertainty as it could introduce new possibilities to more accurately represent the information and help middleware understand the environment. It could be feasible to tackle this issue by extending crisp ontologies with other theories, such as fuzzy logic [63], confidence degree, and uncertainty propagation.

## 5. Conclusions and Future Works

This paper has presented a survey on the robotic intermediation software architecture solutions for underwater environments. A list of robotic middleware, including twelve different candidates which are highly relevant to underwater usages, has been selected from the current literature and projects. This survey differs from other existing survey papers in two distinctive points. On the one hand, it focuses on the study of newest underwater robotic middleware while general purpose robotic middleware proposals are the common scope of interest in other off-the-shelf survey papers. On the other hand, it not only provides a summarization of underwater intermediation solutions with main characteristics explained, but also offers an insightful and comprehensive analysis of those middleware architecture solutions. An assessment of those underwater middleware architectures based on a set of crucial criteria has been carried out. Judging from the study that has been done, it cannot not be ignored that although current underwater middleware solutions have been helpful to some extent to support the cooperation and interaction of different underwater vehicles, none of them offer a performance that includes all the capabilities that are desirable in a software architecture of these characteristics. It can be foreseen that future works about improving current robotic middleware for underwater environments or designing new intermediation solutions from scratch should be emphasized on the following aspects:

- The underwater robotic middleware should fully resolve the heterogeneity inherent to different hardware manufacturers, information exchange and software components so that seamless interconnection and interaction can be achieved to a great extent. A series of ICT technologies, such as the semantic web, cloud computing, and ontologies, can be exploited and reused in underwater robotic middleware.
- The particular constraints coming from tough underwater environments should be considered during the design of underwater robotics middleware. Scarce communication width, an uncertain and noisy environment, heterogeneous data sources and low-capable underwater vehicles will impose a considerable amount of constrains to be tackled by a well-designed underwater robotic middleware.
- The underwater robotic middleware should become innovative by supporting the distributed coordination and integration of information and functionalities coming from different AUVs/ROVs. Improving robustness and reliability of the underwater vehicles could be addressed too. Cost-effectiveness should be guaranteed by leveraging and orchestrating already available functionalities offered by AUVs/ROVs instead of new developments of services from scratch.
- Middleware architectures are expected to have a holistic awareness of the underwater environment so as to adapt its decision/command for vehicles accordingly to the unreliable environment. Context awareness is highly needed and can be realized by an effective and efficient exploitation on data. Semantic capabilities can be added to embed semantics into gathered information, infer more useful knowledge, as well as support the collection and transmission of information to the cooperating AUVs/ROVs involved in specific underwater mission execution process.
- Added value capabilities (*i.e.*, service discovery & registry, fault tolerance, security schemes, friendly user interface) should be encased in the underwater robotic middleware of the future. In this way, the middleware could possibly become suitable for “all settings” in real usages.
- Potential cooperation between different underwater vehicles adopting different underwater robotic middleware architectures should be considered. A major problem hindering the potential cooperation exists in the use of different information models (*i.e.*, ontologies) in different middleware architectures. Ontology alignment or mapping should be addressed to achieve a homogeneous understanding between various middleware architectures.
- The underwater robotic middleware should be tested, validated and demonstrated in relevant and environmentally controlled scenarios under extreme conditions. Furthermore, a step forward trial experiments (*i.e.*, open source licenses or commercial promotion) should be conceived to expand the utilization of underwater robotic middleware intermediation architectures.

The development of an underwater middleware architecture will be among the future works that are going to be carried out by the authors of this manuscript. In order to tackle all the issues that have been highlighted in this manuscript, it will have several key modules that address the features that would be expected:

- One module will be used as an interface with the electronic equipment collecting data from the environment. This module will be responsible for handling heterogeneity regarding robot manufacturers in a deployment.
- Another module will be used to interconnect the middleware architecture to the high level application that make use of the retrieved data.
- There will be other modules that deal with functionalities related to information management: device registration, publish/subscribe paradigm *etc.*
- Finally, more advanced features addressed in this survey, such as security and context awareness will be developed within their own modules. In this way, most of the challenges that have been mentioned here can be solved to a significant extent.
